# Access to a novel first-line single-tablet HIV antiretroviral regimen in Affordable Care Act Marketplace plans, 2018–2020

**DOI:** 10.1186/s40545-023-00559-8

**Published:** 2023-04-20

**Authors:** Rohan Khazanchi, Samuel Powers, Amy Killelea, Andrew Strumpf, Tim Horn, Auntré Hamp, Kathleen A. McManus

**Affiliations:** 1 College of Medicine, University of Nebraska Medical Center, Omaha, NE USA; 2 School of Public Health, University of Minnesota, Minneapolis, MN USA; 3 Division of Infectious Diseases and International Health, Department of Medicine, University of Virginia, P.O. Box 801379, Charlottesville, VA 22908 USA; 4 Health Systems and Policy, National Alliance of State and Territorial AIDS Directors (NASTAD), Washington, DC USA; 5 Present Address: Killelea Consulting, Arlington, VA USA; 6 Present Address: Harvard Internal Medicine-Pediatrics Residency Program, Brigham & Women’s Hospital, Boston Children’s Hospital, and Boston Medical Center, Boston, MA USA; 7 Present Address: Departments of Internal Medicine and Pediatrics, Harvard Medical School, Boston, MA USA; 8 Present Address: FXB Center for Health and Human Rights, Harvard University, Boston, MA USA

**Keywords:** HIV, Access to care, Pharmacoequity, Drug pricing, Coverage, Coinsurance, Copay, Cost

## Abstract

**Background:**

A pillar of the United States’ Ending the HIV Epidemic (EHE) initiative is to rapidly provide antiretroviral therapy (ART) in order to achieve HIV viral suppression. However, insurance benefit design can impede ART access. The primary objective of this study is to understand how Affordable Care Act (ACA) Marketplace qualified health plan (QHP) formularies responded to two new ART single tablet regimens (STRs): dolutegravir/abacavir/lamivudine (DTG/ABC/3TC; approved in 2014) and bictegravir/emtricitabine/tenofovir alafenamide (BIC/FTC/TAF; approved in 2018).

**Methods:**

We conducted a descriptive study of individual and small group QHPs to assess coverage, cost sharing (coinsurance vs. copay), specialty tiering, prior authorization, and out-of-pocket (OOP) costs for DTG/ABC/3TC and BIC/FTC/TAF. All individual and small group QHPs offered in state ACA Marketplaces from 2018–2020 were identified using plan-level formulary data from Ideon linked to end-of-year data from Robert Wood Johnson Foundation’s Individual Market Health Insurance Exchange (HIX).

**Results:**

For 2018, 2019, and 2020, respectively, we identified 19,533, 17,007, and 21,547 QHPs. While DTG/ABC/3TC coverage was above 91% from 2018–2020, BIC/FTC/TAF coverage improved from 60 to 86%. Coverage of BIC/FTC/TAF improved in EHE priority jurisdictions from 73 to 90% driven by increased coverage with coinsurance. Although BIC/FTC/TAF had a higher wholesale acquisition cost than DTG/ABC/3TC, monthly OOP cost trends differed regionally in the Midwest but did not differ by EHE priority jurisdiction status.

**Conclusions:**

QHP coverage of STRs is heterogeneous across the US. While coverage of BIC/FTC/TAF increased over time, many QHPs in EHE priority jurisdictions required coinsurance. Access to new ART regimens may be slowed by delayed QHP coverage and benefit design.

**Supplementary Information:**

The online version contains supplementary material available at 10.1186/s40545-023-00559-8.

## Background

Structural barriers interfere with the United States’ goal of ending the human immunodeficiency virus (HIV) epidemic. When compared with similar high-income countries like the United Kingdom, France, and Canada, the U.S. has the highest antiretroviral therapy (ART) prices and the lowest rate of HIV viral suppression [1]. High ART costs outpace overall inflation rates, with average list prices topping $3,000 per patient per month [2]. “Rapidly and effectively” providing ART to people with HIV (PWH) “to achieve sustained viral suppression,” [3] particularly in Phase I priority jurisdictions, is a key pillar of the U.S. Ending the HIV Epidemic (EHE) plan [4]. Yet, high ART costs are still being passed by insurers onto many PWH [5].

Implemented in 2014, the Patient Protection and Affordable Care Act (ACA) improved access to private insurance for PWH by prohibiting coverage denials based on pre-existing conditions [6, 7]. The ACA also shifted the role of state AIDS Drug Assistance Programs (ADAPs)—state-run safety nets funded through the federal Ryan White HIV/AIDS Program (RWHAP)—to guarantee ART access for uninsured individuals. In recent years, ADAPs have increasingly covered insurance premiums and medication cost sharing (including deductibles, the amount a person pays for health care before insurance starts to pay; copayments, a fixed amount a person pays for healthcare after the deductible has been paid; and coinsurance, a fixed percentage of costs a person pays for healthcare after the deductible has been paid) for their underinsured clients [8]. As almost 90% of ADAP clients had incomes ≤ 250% of the federal poverty level and nearly 45% had no health insurance coverage in 2014 [9], many ADAPs helped enroll clients in subsidized private insurance through ACA Marketplace Qualified Health Plans (QHPs) [10, 11]. Even in Medicaid non-expansion states, ADAP-funded QHP enrollment has been associated with improvement in key HIV outcomes including engagement in care, medication adherence, and viral suppression [11–14].

The ACA’s non-discrimination and coverage requirements mandate essential health benefits. For prescription medications, including ART, QHPs must cover the same number (and at minimum one) of drugs per category/class as a state’s “benchmark” plan [15]. QHPs, in discussion with pharmacy benefit managers, decide which ART regimens to cover, what tier to place them on, and what utilization management to apply. Incentives for these decisions, however, are not always clear and in addition to clinical safety and efficacy considerations could also include discounts and back-end rebates [16, 17]. QHPs have significant flexibility to design their own formularies. As a result, drug coverage varies across QHPs and high cost sharing, specialty drug tiering (a system used by insurance companies to categorize high cost prescription drugs used in the treatment of complex medical conditions, like HIV), and excessive prior authorization requirements (the process of requiring a clinician to obtain approval from a patient’s health insurance prior to treatment) limit ART and HIV pre-exposure prophylaxis access [18–21]. These access barriers may reflect *discriminatory benefit design* where insurance plan characteristics prevent or delay people with complex conditions from obtaining appropriate treatment [18–20]. Evidence of these practices suggests that insurers counterbalanced ACA coverage expansions and pre-existing condition protections with other tactics that dissuade high-cost patients, like PWH, from obtaining or maintaining coverage [20]. Plan-level impediments to ART access may create missed opportunities to achieve HIV viral suppression when transitioning uninsured clients from ADAPs’ largely unrestricted full-pay medication programs to ADAP-funded QHP coverage [22].

Current guidelines for treatment of new HIV-1 infection recommend two nucleos(t)ide reverse transcriptase inhibitors (NRTIs) plus an integrase strand transfer inhibitor (INSTI) [23, 24]. The improved efficacy and safety, minimal toxicity and resistance, and reduced pill burden of INSTI-containing regimens have improved ART adherence [23, 24]. Dolutegravir (an INSTI) was approved by the FDA in 2014 as a once-daily single-tablet regimen (STR) coformulated with lamivudine and abacavir (DTG/ABC/3TC). Bictegravir (a newer INSTI) was approved by the FDA in 2018 as a once-daily STR coformulated with emtricitabine and tenofovir alafenamide (BIC/FTC/TAF). In a double-blind, multicenter, phase 3, randomized controlled trial of treatment-naive PWH, BIC/FTC/TAF was non-inferior to DTG/ABC/3TC in achieving viral suppression, had better gastrointestinal tolerability, and had no evidence of treatment-emergent resistance at 48 weeks [25, 26]. BIC/FTC/TAF is also easier to swallow and perceived as smaller by ART-naïve PWH than DTG/ABC/3TC, which may mitigate suboptimal adherence due to size-related dysphagia [27, 28]. In 2020, the monthly wholesale acquisition cost (WAC) for BIC/FTC/TAF was $3,238.31 while the WAC for DTG/ABC/3TC was $3,032.09 [29].

Rapid ART initiation shortly after diagnosis improves uptake, linkage to care, and time to viral suppression [26, 30–35] even among populations facing significant structural vulnerabilities in the U.S [26, 35] and abroad [33, 34]. With this in mind, expert guidelines now recommend ART initiation immediately upon diagnosis in amenable PWH who do not have opportunistic infections [23, 24]. Since BIC/FTC/TAF does not contain abacavir, HLA B*5701 testing results are not immediately required for prescription, making it an ideal option for same-day ART initiation [23].

A 2016 evaluation of ACA QHPs revealed discriminatory benefit designs which may discourage PWH from enrolling in QHPs or adhering to STRs like DTG/ABC/3TC [18]. However, studies have yet to evaluate QHP coverage since the introduction of BIC/FTC/TAF or shifts in coverage of older STRs in the wake of a new competitor. In this study, we investigated whether there were differences in 2018–2020 QHP characteristics (coverage, cost sharing structure, specialty tiering, prior authorization requirements, and out-of-pocket [OOP] costs) by state, region, and EHE Phase I jurisdiction status for BIC/FTC/TAF versus DTG/ABC/3TC. We hypothesized that due to it being newer and more expensive, BIC/FTC/TAF would have lower prevalence of coverage, higher cost sharing, more frequent specialty tiering and prior authorization requirements, and higher OOP costs than DTG/ABC/3TC, but that benefit design would improve throughout the study period. We also hypothesized that there may be more restrictive coverage in the South and in EHE priority jurisdictions, given prior evidence of regionalized differences in access to HIV prevention medications through QHPs [21].

## Methods

### Data

This cross-sectional study used 2018–2020 end-of-year data from the Robert Wood Johnson Foundation’s publicly-available Individual Market Health Insurance Exchange (HIX) Compare linked with 2018–2020 plan-level formulary data from Ideon. We received data from Ideon through a data use agreement. We assessed plan-specific variables for the two most recommended STRs for HIV (DTG/ABC/3TC and BIC/FTC/TAF). This study followed the Strengthening the Reporting of Observational Studies in Epidemiology (STROBE) reporting guideline.

We defined a unique QHP as an ACA-compliant individual plan offered on the ACA Marketplace in a specific rating area. Plans offered in the same state with the same cost sharing benefits in different rating areas were considered different plans. Cost sharing reduction and child-only variants of QHPs were not included.

### Variables

We investigated plan characteristics (coverage, cost sharing structure, tiering, prior authorization requirement, monthly OOP cost) nationwide and for each census region (Northeast, West, Midwest, and South), EHE Phase I jurisdiction status, and state. EHE Phase I jurisdictions include counties that accounted for more than half of new HIV diagnoses in 2016 and 2017, and seven states with a substantial rural HIV burden [4]. Drugs were coded as “not covered” if they did not appear on the formulary or if the formulary classified them as “not listed” or “not covered”. For each drug covered by a plan, cost sharing was categorized as either “copay” (payment based on a predetermined rate) or “coinsurance” (payment based on a percentage of the total medication list price), prior authorization was “required” or “not required”, drug tiering was categorized as “specialty” or “non-specialty”, and average monthly OOP drug cost was estimated by applying relevant plan characteristics including copay, coinsurance, deductible, and OOP maximum to the monthly wholesale acquisition cost (WAC). We reported monthly OOP costs as “costs of access”, calculated from the OOP cost of QHP premiums plus the OOP drug cost for DTG/ABC/3TC or BIC/FTC/TAF.

### Statistical analysis

We calculated descriptive statistics for variables of interest at national, regional, and state levels and by EHE Phase I jurisdiction status. We did not report statistical significance tests, given that this study reports trends which encompass the entire population of ACA QHPs. We analyzed data using R Statistical Software (R Foundation for Statistical Computing), version 4.0.2, and RStudio.

## Results

For the years 2018, 2019, and 2020, respectively, we identified 19,533, 17,007, and 21,547 unique QHPs with formulary data for DTG/ABC/3TC or BIC/FTC/TAF.

### Coverage and cost sharing structure

#### National and regional

Overall, 93% of QHPs covered DTG/ABC/3TC and 60% covered BIC/FTC/TAF in 2018. DTG/ABC/3TC coverage improved to 97% in 2019 and decreased to 91% in 2020, whereas BIC/FTC/TAF coverage decreased slightly to 59% in 2019 and increased to 86% in 2020.

**Fig. 1:**
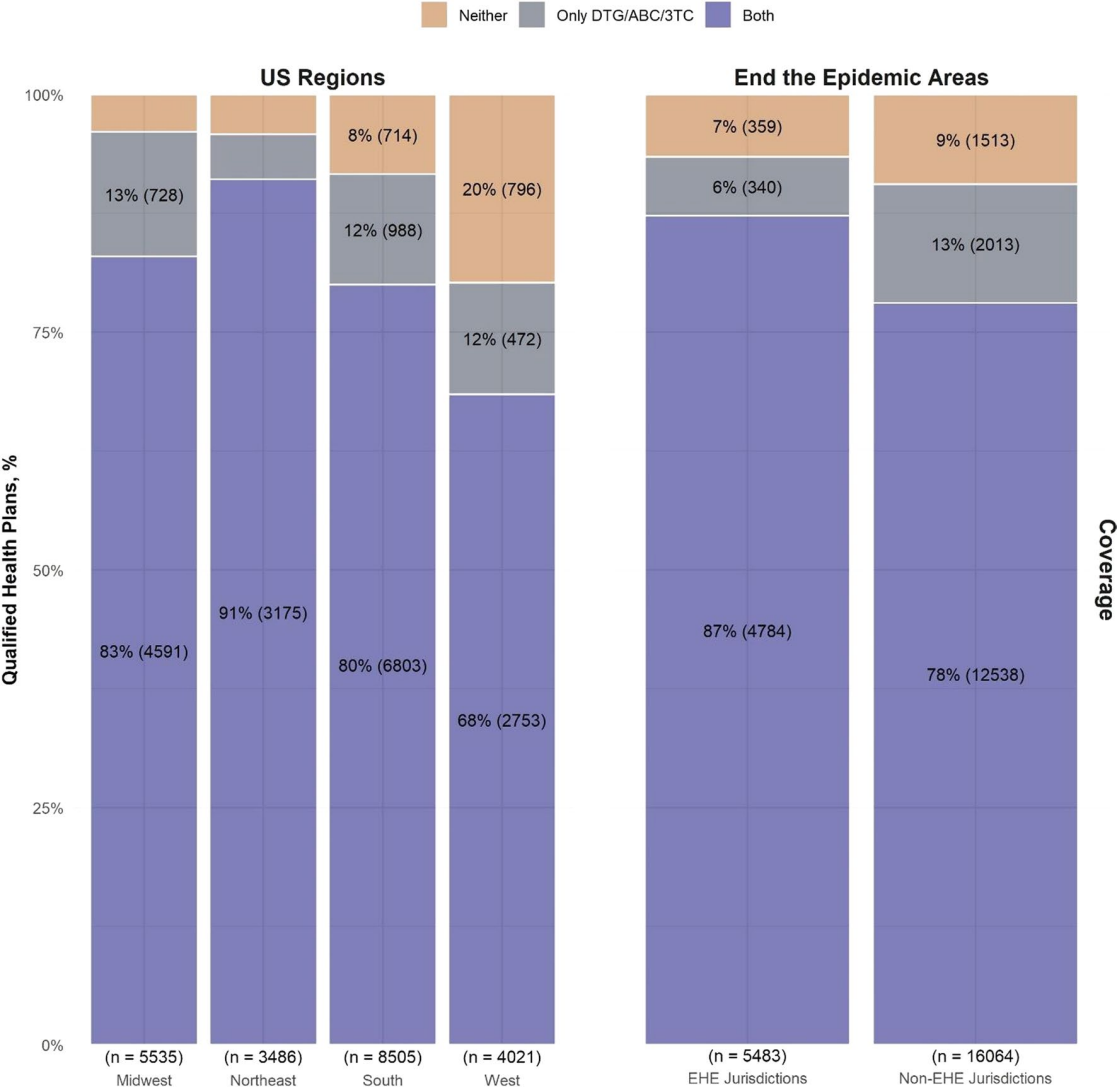
Coverage of DTG/ABC/3TC and BIC/FTC/TAF by Region and “Ending the HIV Epidemic” Status, 2020. Coverage of both medications was higher in the Northeast, compared to the Midwest, South and West. Coverage of both medications was lower among QHPs in the West compared to the other regions. A higher proportion of QHPs in EHE jurisdictions covered both medications compared to non-EHE jurisdictions

More QHPs offered coverage of both medications in the Northeast (91%) than other regions (Fig. 1). Coverage of DTG/ABC/3TC was nearly ubiquitous across all regions other than the West and remained stable. In contrast, coverage of BIC/FTC/TAF improved from 2019 to 2020 across all regions and especially the Midwest (31% to 88%). However, prevalence of BIC/FTC/TAF coverage remained below DTG/ABC/3TC coverage throughout the study period and across all four regions.

**Fig. 2:**
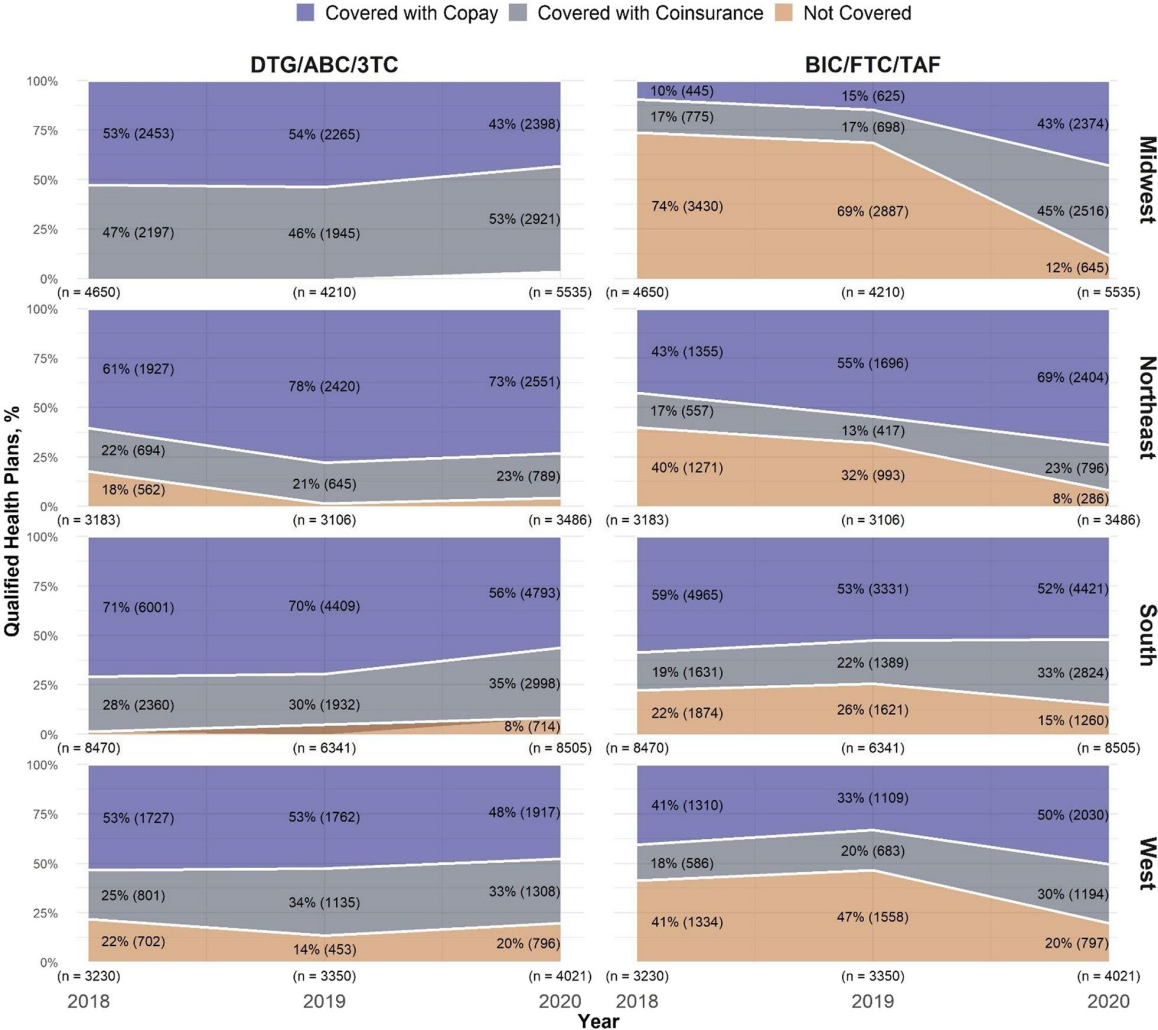
Qualified Health Plan Coverage and Cost Sharing for DTG/ABC/3TC and BIC/FTC/TAF by Region, 2018–2020. Coverage of BIC/FTC/TAF increased in each of the regions, driven by coverage with coinsurance. DTG/ABC/3TC coverage was stable for QHPs in the Midwest, decreased slightly among the South, and increased for Northeast and West states

A larger proportion of QHPs required coinsurance for BIC/FTC/TAF compared to DTG/ABC/3TC across all four regions throughout the study period (Fig. 2). From 2018 to 2020 in the South, the proportion covering BIC/FTC/TAF with copay decreased (59% to 52%) while the proportion of QHPs requiring coinsurance increased (19% to 33%). In contrast, the proportion of QHPs covering BIC/FTC/TAF with copay increased in all other regions.

#### EHE

**Fig. 3:**
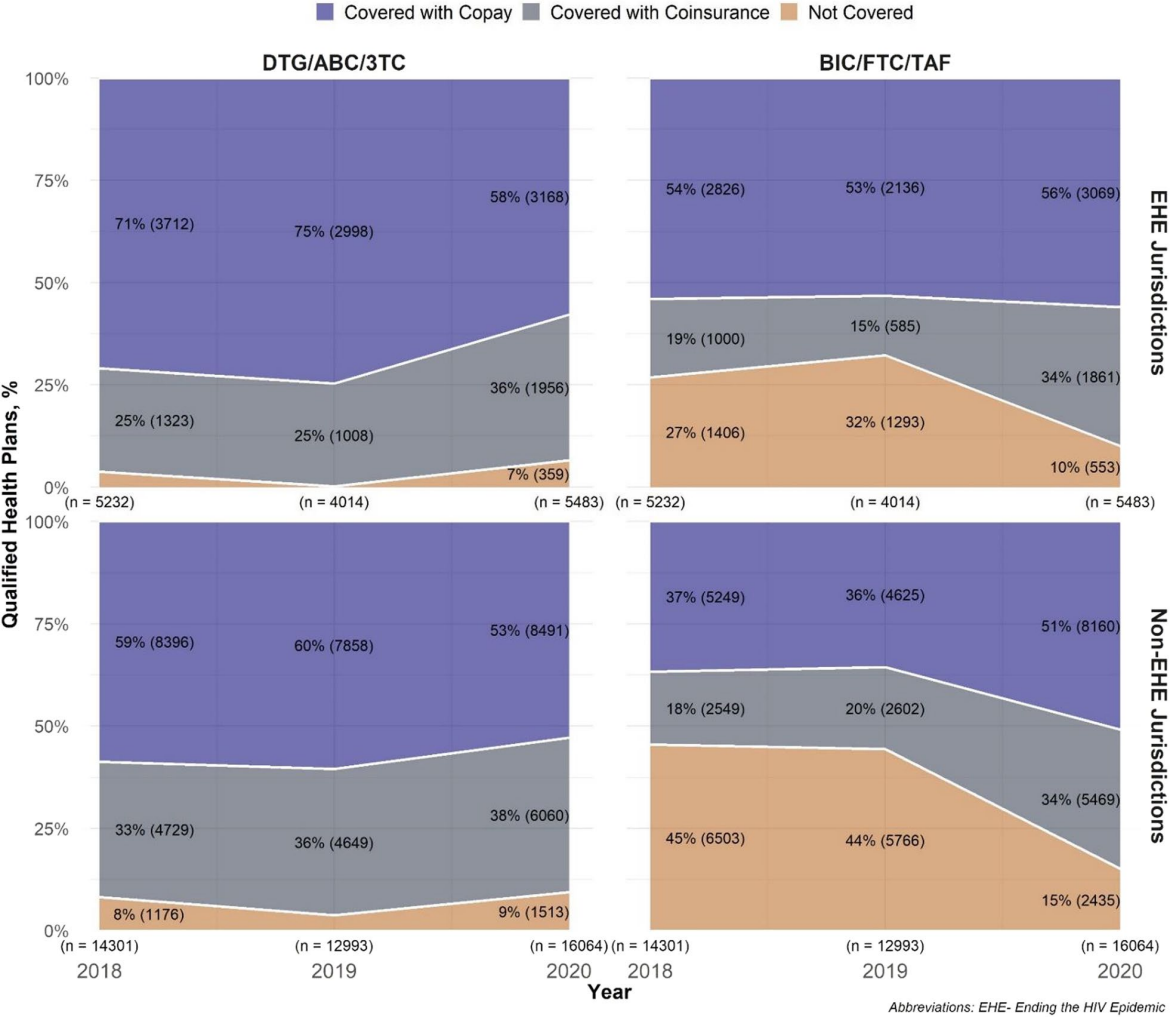
Qualified Health Plan Coverage for DTG/ABC/3TC and BIC/FTC/TAF by “Ending the HIV Epidemic” Status, 2018–2020. Coverage of BIC/FTC/TAF increased in both EHE and non-EHE jurisdictions, driven by coverage with coinsurance

From 2018 to 2020, we identified 5232, 4014, and 5483 unique QHPs in EHE Phase I priority jurisdictions and 14,301, 12,993, and 16,064 unique QHPs in non-EHE jurisdictions. In 2020, more QHPs covered both medications in EHE (87%) than non-EHE jurisdictions (78%; Fig. 1). Overall, QHP coverage of DTG/ABC/3TC was better than coverage of BIC/FTC/TAF in both EHE and non-EHE jurisdictions and across all 3 years (Fig. 3). By 2020, BIC/FTC/TAF coverage was higher in EHE jurisdictions (90%) compared to non-EHE (85%). Increases in BIC/FTC/TAF coverage in EHE jurisdictions from 74% in 2018 to 90% in 2020 were mostly driven by increased coverage with coinsurance (19% to 34%); the proportion of QHPs covering BIC/FTC/TAF with copay increased slightly (54% to 56%).

#### State

**Fig. 4:**
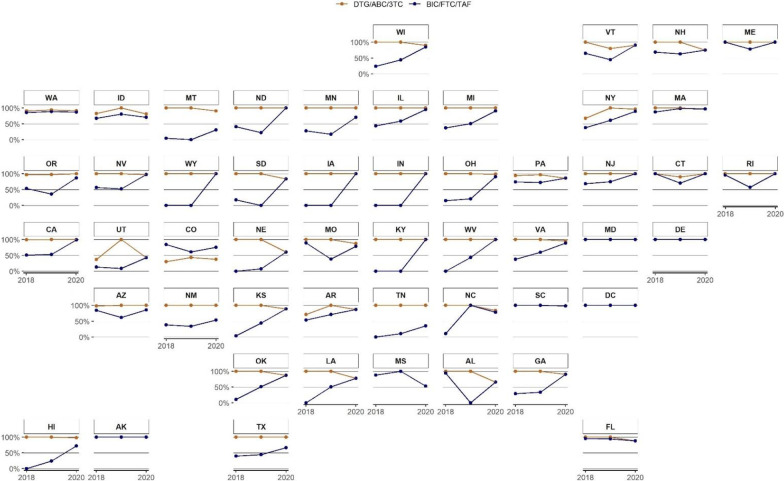
Qualified Health Plan Coverage of DTG/ABC/3TC and BIC/FTC/TAF by State, 2018–2020. For each state, the y-axis reflects the proportion of qualified health plans offering coverage for either DTG/ABC/3TC and BIC/FTC/TAF. Coverage of both medications was consistently high in states like MD, DE, SC, and DC. Coverage of BIC/FTC/TAF increased in most states except for MS, ID, SC, and FL. The point values for each state-year are provided in Additional file 1: Table S1

Figure 4 illustrates state-by-state trends in coverage of DTG/ABC/3TC and BIC/FTC/TAF from 2018 to 2020 (see table, Additional File 1 for individual values by state-year). In 2018, 26 states had < 50% of QHPs covering BIC/FTC/TAF, and nine states (WY, WV, TN, NE, LA, KY, IN, IA, HI) had zero QHPs offering BIC/FTC/TAF coverage. In contrast, 41 states covered DTG/ABC/3TC in 100% of QHPs, and only Colorado and Utah had < 50% of QHPs covering DTG/ABC/3TC. These differences persisted in 2019, with BIC/FTC/TAF coverage decreasing in 11 states (ND, SD, MN, AL, OR, AZ, CT, RI, VT, ME, MO). Though coverage of BIC/FTC/TAF approached DTG/ABC/3TC levels in 2020, the majority of states still offered more QHPs covering DTG/ABC/3TC. Changes in coverage with copay vs. coinsurance by state are provided in Additional file 2a, b.

### Tiering

Nationally, the prevalence of specialty tiering was similar for both medications, with higher rates for DTG/ABC/3TC compared to BIC/FTC/TAF (14%, 18%, 19% vs. 9%, 13%, 19% for 2018, 2019, and 2020, respectively). Prevalence of specialty and non-specialty drug tiering are provided by region, EHE priority status, and state in Additional file 3a, b. Across all three years, QHPs in EHE Phase I priority jurisdictions were less likely to require specialty drug tiering than non-EHE jurisdictions (DTG/ABC/3TC: EHE 6%, 10%, 9% vs. non-EHE 17%, 21%, 23%; BIC/FTC/TAF: EHE 2%, 4%, 9% vs. non-EHE 12%, 16%, 23% for 2018, 2019, and 2020, respectively).

### Prior authorization (PA) requirement

Overall, the prevalence of PA requirements for QHPs covering DTG/ABC/3TC remained low throughout the study period (2%, 2%, 1% for 2018, 2019, and 2020, respectively). PA requirements for BIC/FTC/TAF were more frequent at 5% in 2018 and 8% in 2019, but were eliminated in 2020 by all QHPs except 18 plans (5% of QHPs statewide) in Washington.

The prevalence of PA requirements is provided by region, EHE priority status, and state in Additional file 4. Regionally, PA for DTG/ABC/3TC was required in 1% of QHPs in the Northeast by 2020 (from 4 and 3% in 2018 and 2019, respectively). The West and South had zero QHPs requiring PA for DTG/ABC/3TC. The Midwest similarly maintained low prevalence of PA for DTG/ABC/3TC (3%, 4%, and 1% for 2018, 2019, and 2020, respectively), with QHPs requiring PA mostly concentrated in Michigan where ~ 20% of QHPs required PA from 2018–2019. In 2018 and 2019, PA requirements for BIC/FTC/TAF were most prevalent in the Northeast (19% and 34%) compared to the West (2%, 6%), Midwest (4%, 0%), and South (3%, 0%). This was driven by a majority of plans in New York requiring PA (357 QHPs [55% of QHPs statewide] and 705 [62%] in 2018 and 2019, respectively).

### Monthly OOP cost of access

Nationally, the costs of access to both drugs were very similar throughout the study period and across cost sharing types (see table, Additional file 5, for national costs). For both drugs, higher costs of access were associated with plans that used coinsurance (DTC/ABC/3TC: copay $681, $737, $739 vs. coinsurance $958, $1002, $1008; BIC/FTC/TAF copay $696, $744, $733 vs. coinsurance $945, $1003, $1012 for 2018, 2019, and 2020, respectively).

Average monthly costs of access for each drug from 2018 to 2020 are provided by region, EHE priority status, and state in Additional file 5. When stratified by cost sharing type and region, costs did not differ substantively except among QHPs with copay in the Midwest. In this subset of Midwest QHPs, BIC/FTC/TAF had a higher OOP cost than DTG/ABC/3TC in 2018 but trended below the cost of DTG/ABC/3TC by 2020. Costs of access did not differ substantively by EHE Phase I priority jurisdiction status.

## Discussion

Our findings highlight that QHPs may impede uptake of novel HIV STRs through non-coverage decisions and plan designs that limit access, especially in the first year after approval. At the end of 2018, the year when BIC/FTC/TAF was introduced, fewer than half of QHPs in 26 states and zero QHPs in nine states offered coverage. In contrast, DTG/ABC/3TC coverage was nearly ubiquitous across all but two states. Over time, coverage of BIC/FTC/TAF increased, but improvements in EHE priority jurisdictions and the South were largely driven by increased coverage using coinsurance. OOP costs associated with coinsurance tend to be higher than costs associated with copays since coinsurance cost is based on a percentage of medication list price; thus, coverage expansions with coinsurance may be less desirable for ART regimens. For BIC/FTC/TAF, prior authorization requirements were alarmingly prevalent in NY from 2018–2019 but dissipated by 2020 and were generally similar to DTG/ABC/3TC elsewhere. Specialty tiering prevalence for BIC/FTC/TAF peaked in several Midwest states from 2018–2019 but was otherwise generally similar to DTG/ABC/3TC. Overall, QHP benefit designs may have interfered with clinicians’ ability to use a novel first-line STR for rapid ART initiation—leaving PWH waiting for plans to settle into benefit designs that did not limit access.

Despite a $190 greater monthly wholesale acquisition cost (WAC) for BIC/FTC/TAF compared to DTG/ABC/3TC in 2020 [29], we found that monthly OOP costs were not meaningfully different when stratified by cost sharing type, region, or EHE jurisdiction status. However, both medications consistently had $700–$1000 monthly OOP costs and a $2700 + monthly WAC [29]; this should still be reason for alarm. Drug list prices are significant drivers of coverage delays and utilization management, which can limit access to new regimens and shift financial burdens onto patients [36]. Moreover, prescription drug costs have stark implications for PWH, as cost-related medication nonadherence remains prevalent and is associated with worse HIV care engagement, lower likelihood of achieving viral suppression, increased rates of emergency department use and hospitalization, and increased overall costs to the health care system [37–39]. Even though “Treatment as Prevention” through adherence to ART is both the most clinically effective and cost-effective intervention to reduce incidence of new HIV cases [40, 41], our findings reaffirm that the U.S. EHE plan has yet to address high and growing ART prices, which far outpace annual inflation [2]. Although ADAPs can assist QHP-enrolled PWH with OOP costs and discount ART regimens through the 340B Drug Pricing Program and direct negotiation with manufacturers, the growing list prices of prescription drugs may impact the continued financial sustainability of ADAPs.

Situated in the broader context of HIV therapeutic development, our findings may have implications for the future of HIV care. Long-acting injectable ART formulations combining cabotegravir and ripilvirine received U.S. Food and Drug Administration approval in January 2021 after demonstrating efficacy and safety in pivotal international, phase 3, randomized, controlled trials [42–44]. These regimens may be especially efficacious for individuals facing social, structural, and behavioral barriers which challenge their adherence to daily oral therapy (e.g., individuals with multiple prior treatment failures) [45, 46]. With these promising developments on the horizon, ADAPs’ ability to afford drugs on behalf of clients or to assist with insured clients’ cost sharing remains a key consideration in whether PWH will or will not easily gain access to novel therapeutics like long-acting injectable ART [47–49].

Overall, our analysis affirms that targeted interventions may be necessary to ensure QHPs have adequate benefit design and offer coverage of U.S. Department of Health and Human Services guideline-recommended first-line HIV therapeutics. We also recognize that, though beyond the scope of our analysis, these interventions should consider the intertwined roles of manufacturers, pharmacy benefit managers (PBMs), and insurance plans in exacerbating high drug prices [50]. Multi-level interventions could involve directly lowering manufacturer prices through government regulation and price negotiation; incentivizing PBMs to negotiate lower list prices while limiting overreliance on manufacturer rebates; incentivizing favorable drug tiering by insurance plans; disentangling prescription drug costs from specialty drug tiers; basing coinsurance-associated OOP costs on post-discounted rather than pre-discounted drug prices, to pass negotiated discounts down to PWH; and ensuring QHPs rapidly expand coverage with reasonable cost sharing structures for any novel, efficacious, and cost-effective therapeutics [36, 50, 51]. PWH enrolled in ADAP-funded QHPs were more likely to achieve viral suppression than PWH receiving their medications directly from state ADAPs [11–14]. Even still, ADAPs continue to face precarious funding streams routed through annual federal allocations and discretionary state support [52]. Regulatory interventions within the pharmaceutical and insurance industries, like those aforementioned, may be critical to continue the financial sustainability and potential clinical benefits of ADAP-funded QHP enrollment.

### Strengths and limitations

Strengths of this work include our comprehensive, national scope, achieved by analyzing multiple characteristics of all ACA Marketplace insurance plans across all U.S. states. Limitations include that our measure of OOP cost does not account for some ART-associated costs, including HIV medical care visits and ART-related laboratory monitoring, though these additional expenses are small relative to the high cost of ART regimens. Finally, we were unable to interrogate the full spectrum of structural factors impacting medication coverage and cost due to data unavailability regarding PBM rebates and other intermediary steps. This work did not study the clustering of specific utilization management techniques for STR ART regimens. Future work should determine if there are groups of more or less restrictive plans for specific STR ART regimens or STR ART regimens in general.

## Conclusions

Our findings highlight how initial access to novel HIV therapeutics may be constrained by variability in insurance coverage and plan characteristics. These constraints are prevalent and heterogeneous at state and regional levels, as well as among high-priority jurisdictions identified by the U.S. “Ending the HIV Epidemic” strategy. As novel HIV therapeutics emerge and the EHE initiative’s “Treatment as Prevention” arm grows, state and federal policy interventions should consider the roles of insurance benefit design, drug prices, and high OOP costs in limiting equitable access to first-line HIV ART regimens.

## Supplementary Information

**Additional file 1.** Overall QHP Coverage of DTG/ABC/3TC and BIC/FTC/TAF by Census Region, EHE Jurisdiction, and State, 2018–2020.

**Additional file 2. **Cost Sharing Structure for QHPs Coverage of DTG/ABC/3TC by Census Region, EHE Jurisdiction, and State, 2018–2020. Cost Sharing Structure for QHP Coverage of BIC/FTC/TAF by Census Region, EHE Jurisdiction, and State, 2018–2020.

**Additional file 3.** Drug Tiering for QHP Coverage of DTG/ABC/3TC by Census Region, EHE Jurisdiction, and State, 2018–2020. Drug Tiering for QHP Coverage of BIC/FTC/TAF by Census Region, EHE Jurisdiction, and State, 2018–2020.

**Additional file 4.** Prior Authorization Requirement for DTG/ABC/3TC and BIC/FTC/TAF by Census Region, EHE Jurisdiction, and State, 2018–2020.

**Additional file 5.** Average Out-of-Pocket (OOP) Cost per Month for QHP Coverage of DTG/ABC/3TC and BIC/FTC/TAF by U.S., Census Region, EHE Jurisdiction, and State, 2018–2020.

## Data Availability

The data that support the findings of this study include publicly-available data from Robert Wood Johnson’s HIX Compare and data received from Ideon under a data use agreement. The data use agreement precludes our sharing of the data. Interested parties are referred to these two avenues to access the data.

## Abbreviations

EHE: Ending the HIV Epidemic
ART: Antiretroviral therapy
ACA: Affordable Care Act
QHP: Qualified health plan
STRs: Single tablet regimens
DTG/ABC/3TC: Dolutegravir/abacavir/lamivudine
BIC/FTC/TAF: Bictegravir/emtricitabine/tenofovir alafenamide
HIX: Health Insurance Exchange
PWH: People with HIV
HIV: Human Immunodeficiency Virus
ADAPs: AIDS Drug Assistance Programs
RWHAP: Ryan White HIV/AIDS Program
NRTI: Nucleos(t)ide reverse transcriptase inhibitors
INSTI: Integrase strand transfer inhibitor
PA: Prior authorization
WAC: Wholesale acquisition cost
PBM: Pharmacy benefit manager
