# Supplementary material for: Access to a novel first-line single-tablet HIV antiretroviral regimen in Affordable Care Act Marketplace plans, 2018–2020
Source: J Pharm Policy Pract. 2023 Apr 20;16:57. doi: 10.1186/s40545-023-00559-8 (PMC10116786; doi:10.1186/s40545-023-00559-8)
Supplement: Supplementary file 1 — Additional file 1. Overall QHP Coverage of DTG/ABC/3TC and BIC/FTC/TAF by Census Region, EHE Jurisdiction, and State, 2018–2020. [file 40545_2023_559_MOESM1_ESM.docx]

**Additional File 1.** Overall Coverage of DTG/ABC/3TC and BIC/FTC/TAF by Census Region, EHE Jurisdiction, and State, 2018 – 2020

|  | **DTG/ABC/3TC** | | | | | | | | | | **BIC/FTC/TAF** | | | | | | | | |
| --- | --- | --- | --- | --- | --- | --- | --- | --- | --- | --- | --- | --- | --- | --- | --- | --- | --- | --- | --- |
|  | **2018** | | | **2019** | | | **2020** | | | **2018** | | | | **2019** | | | **2020** | | |
| Characteristics | total plans | covered | % | total plans | covered | % | total plans | covered | % | total plans | | covered | % | total plans | covered | % | total plans | covered | % |
| National | 19533 | 18160 | 93 | 17007 | 16513 | 97 | 21547 | 19675 | 91 | 19533 | | 11624 | 60 | 17007 | 9948 | 58 | 21547 | 18559 | 86 |
| Regional |  |  |  |  |  |  |  |  |  |  | |  |  |  |  |  |  |  |  |
| Northeast | 3183 | 2621 | 82 | 3106 | 3065 | 99 | 3486 | 3340 | 96 | 3183 | | 1912 | 60 | 3106 | 2133 | 69 | 3486 | 3200 | 92 |
| Midwest | 4650 | 4650 | 100 | 4210 | 4210 | 100 | 5535 | 5319 | 96 | 4650 | | 1220 | 26 | 4210 | 1323 | 31 | 5535 | 4890 | 88 |
| South | 8470 | 8361 | 99 | 6341 | 6341 | 100 | 8505 | 7791 | 92 | 8470 | | 6596 | 78 | 6341 | 4720 | 74 | 8505 | 7245 | 85 |
| West | 3230 | 2528 | 78 | 3350 | 2897 | 86 | 4021 | 3225 | 80 | 3230 | | 1896 | 59 | 3350 | 1792 | 53 | 4021 | 3224 | 80 |
| EHE Status |  |  |  |  |  |  |  |  |  |  | |  |  |  |  |  |  |  |  |
| EHE | 5232 | 5035 | 97 | 4014 | 4006 | 100 | 5483 | 5124 | 93 | 5232 | | 3826 | 73 | 4014 | 2721 | 68 | 5483 | 4930 | 90 |
| Non-EHE | 14301 | 13125 | 92 | 12993 | 12507 | 96 | 16064 | 14551 | 91 | 14301 | | 7798 | 55 | 12993 | 7227 | 56 | 16064 | 13629 | 85 |
| State |  |  |  |  |  |  |  |  |  |  | |  |  |  |  |  |  |  |  |
| AK | 15 | 15 | 100 | 15 | 15 | 100 | 26 | 26 | 100 | 15 | | 15 | 100 | 15 | 15 | 100 | 26 | 26 | 100 |
| AL | 138 | 138 | 100 | 97 | 97 | 100 | 138 | 91 | 66 | 138 | | 130 | 94 | 97 | 0 | 0 | 138 | 91 | 66 |
| AR | 299 | 214 | 72 | 167 | 167 | 100 | 217 | 189 | 87 | 299 | | 161 | 54 | 167 | 119 | 71 | 217 | 189 | 87 |
| AZ | 65 | 64 | 98 | 107 | 107 | 100 | 205 | 205 | 100 | 65 | | 55 | 85 | 107 | 66 | 62 | 205 | 176 | 86 |
| CA | 995 | 994 | 100 | 1024 | 1024 | 100 | 1087 | 1087 | 100 | 995 | | 511 | 51 | 1024 | 545 | 53 | 1087 | 1075 | 99 |
| CO | 711 | 216 | 30 | 768 | 336 | 44 | 817 | 310 | 38 | 711 | | 602 | 85 | 768 | 473 | 62 | 817 | 617 | 76 |
| CT | 256 | 256 | 100 | 248 | 224 | 90 | 272 | 272 | 100 | 256 | | 256 | 100 | 248 | 176 | 71 | 272 | 272 | 100 |
| DC | 26 | 26 | 100 | 25 | 25 | 100 | 25 | 25 | 100 | 26 | | 26 | 100 | 25 | 25 | 100 | 25 | 25 | 100 |
| DE | 7 | 7 | 100 | 8 | 8 | 100 | 11 | 11 | 100 | 7 | | 7 | 100 | 8 | 8 | 100 | 11 | 11 | 100 |
| FL | 3931 | 3931 | 100 | 2336 | 2336 | 100 | 3274 | 2881 | 88 | 3931 | | 3757 | 96 | 2336 | 2196 | 94 | 3274 | 2881 | 88 |
| GA | 322 | 322 | 100 | 545 | 545 | 100 | 571 | 516 | 90 | 322 | | 94 | 29 | 545 | 184 | 34 | 571 | 516 | 90 |
| HI | 44 | 44 | 100 | 45 | 45 | 100 | 46 | 45 | 98 | 44 | | 0 | 0 | 45 | 11 | 24 | 46 | 33 | 72 |
| IA | 84 | 84 | 100 | 141 | 141 | 100 | 160 | 160 | 100 | 84 | | 0 | 0 | 141 | 0 | 0 | 160 | 160 | 100 |
| ID | 380 | 315 | 83 | 319 | 319 | 100 | 326 | 266 | 82 | 380 | | 257 | 68 | 319 | 257 | 81 | 326 | 231 | 71 |
| IL | 323 | 323 | 100 | 278 | 278 | 100 | 331 | 331 | 100 | 323 | | 140 | 43 | 278 | 163 | 59 | 331 | 316 | 95 |
| IN | 440 | 440 | 100 | 375 | 375 | 100 | 528 | 528 | 100 | 440 | | 0 | 0 | 375 | 0 | 0 | 528 | 528 | 100 |
| KS | 95 | 95 | 100 | 90 | 90 | 100 | 191 | 170 | 89 | 95 | | 4 | 4 | 90 | 40 | 44 | 191 | 170 | 89 |
| KY | 142 | 142 | 100 | 133 | 133 | 100 | 133 | 133 | 100 | 142 | | 0 | 0 | 133 | 0 | 0 | 133 | 133 | 100 |
| LA | 206 | 206 | 100 | 203 | 203 | 100 | 199 | 154 | 77 | 206 | | 0 | 0 | 203 | 103 | 51 | 199 | 154 | 77 |
| MA | 591 | 591 | 100 | 328 | 328 | 100 | 457 | 443 | 97 | 591 | | 519 | 88 | 328 | 323 | 98 | 457 | 443 | 97 |
| MD | 152 | 152 | 100 | 108 | 108 | 100 | 128 | 128 | 100 | 152 | | 152 | 100 | 108 | 108 | 100 | 128 | 128 | 100 |
| ME | 94 | 94 | 100 | 227 | 227 | 100 | 284 | 284 | 100 | 94 | | 94 | 100 | 227 | 177 | 78 | 284 | 284 | 100 |
| MI | 833 | 833 | 100 | 867 | 867 | 100 | 938 | 938 | 100 | 833 | | 312 | 37 | 867 | 437 | 50 | 938 | 851 | 91 |
| MN | 366 | 366 | 100 | 389 | 389 | 100 | 501 | 501 | 100 | 366 | | 102 | 28 | 389 | 66 | 17 | 501 | 354 | 71 |
| MO | 193 | 193 | 100 | 161 | 161 | 100 | 239 | 209 | 87 | 193 | | 173 | 90 | 161 | 62 | 39 | 239 | 189 | 79 |
| MS | 198 | 174 | 100 | 30 | 30 | 100 | 102 | 54 | 53 | 198 | | 174 | 88 | 30 | 30 | 100 | 102 | 54 | 53 |
| MT | 86 | 86 | 100 | 102 | 102 | 100 | 128 | 116 | 91 | 86 | | 4 | 5 | 102 | 0 | 0 | 128 | 40 | 31 |
| NC | 164 | 164 | 100 | 192 | 192 | 100 | 251 | 211 | 84 | 164 | | 18 | 11 | 192 | 192 | 100 | 251 | 196 | 78 |
| ND | 88 | 88 | 100 | 125 | 125 | 100 | 153 | 153 | 100 | 88 | | 36 | 41 | 125 | 28 | 22 | 153 | 153 | 100 |
| NE | 42 | 42 | 100 | 51 | 51 | 100 | 115 | 70 | 61 | 42 | | 0 | 0 | 51 | 4 | 8 | 115 | 70 | 61 |
| NH | 35 | 35 | 100 | 27 | 27 | 100 | 36 | 27 | 75 | 35 | | 24 | 69 | 27 | 17 | 63 | 36 | 27 | 75 |
| NJ | 155 | 155 | 100 | 28 | 28 | 100 | 29 | 29 | 100 | 155 | | 107 | 69 | 28 | 21 | 75 | 29 | 29 | 100 |
| NM | 168 | 168 | 100 | 205 | 205 | 100 | 195 | 195 | 100 | 168 | | 65 | 39 | 205 | 70 | 34 | 195 | 105 | 54 |
| NV | 189 | 189 | 100 | 38 | 38 | 100 | 180 | 176 | 98 | 189 | | 107 | 57 | 38 | 20 | 53 | 180 | 176 | 98 |
| NY | 1699 | 1153 | 68 | 1855 | 1855 | 100 | 1877 | 1817 | 97 | 1699 | | 646 | 38 | 1855 | 1133 | 61 | 1877 | 1677 | 89 |
| OH | 807 | 807 | 100 | 888 | 888 | 100 | 1315 | 1305 | 99 | 807 | | 125 | 15 | 888 | 185 | 21 | 1315 | 1196 | 91 |
| OK | 47 | 47 | 100 | 107 | 107 | 100 | 153 | 133 | 87 | 47 | | 5 | 11 | 107 | 55 | 51 | 153 | 133 | 87 |
| OR | 188 | 183 | 97 | 199 | 194 | 97 | 279 | 279 | 100 | 188 | | 101 | 54 | 199 | 72 | 36 | 279 | 243 | 87 |
| PA | 285 | 269 | 95 | 301 | 292 | 97 | 431 | 372 | 86 | 285 | | 213 | 75 | 301 | 218 | 72 | 431 | 372 | 86 |
| RI | 28 | 28 | 100 | 52 | 52 | 100 | 56 | 56 | 100 | 28 | | 27 | 96 | 52 | 30 | 58 | 56 | 56 | 100 |
| SC | 1748 | 1748 | 100 | 1117 | 1117 | 100 | 1467 | 1440 | 98 | 1748 | | 1748 | 100 | 1117 | 1117 | 100 | 1467 | 1440 | 98 |
| SD | 102 | 102 | 100 | 82 | 82 | 100 | 98 | 82 | 84 | 102 | | 18 | 18 | 82 | 0 | 0 | 98 | 82 | 84 |
| TN | 44 | 44 | 100 | 92 | 92 | 100 | 187 | 187 | 100 | 44 | | 0 | 0 | 92 | 10 | 11 | 187 | 66 | 35 |
| TX | 576 | 576 | 100 | 698 | 698 | 100 | 1166 | 1166 | 100 | 576 | | 228 | 40 | 698 | 311 | 45 | 1166 | 776 | 67 |
| UT | 190 | 71 | 37 | 225 | 225 | 100 | 314 | 135 | 43 | 190 | | 26 | 14 | 225 | 20 | 9 | 314 | 135 | 43 |
| VA | 254 | 254 | 100 | 307 | 307 | 100 | 285 | 274 | 96 | 254 | | 96 | 38 | 307 | 185 | 60 | 285 | 254 | 89 |
| VT | 40 | 40 | 100 | 40 | 32 | 80 | 44 | 40 | 91 | 40 | | 26 | 65 | 40 | 18 | 45 | 44 | 40 | 91 |
| WA | 178 | 162 | 91 | 273 | 257 | 94 | 388 | 355 | 91 | 178 | | 153 | 86 | 273 | 243 | 89 | 388 | 337 | 87 |
| WI | 1277 | 1277 | 100 | 763 | 763 | 100 | 966 | 872 | 90 | 1277 | | 310 | 24 | 763 | 338 | 44 | 966 | 821 | 85 |
| WV | 216 | 216 | 100 | 176 | 176 | 100 | 198 | 198 | 100 | 216 | | 0 | 0 | 176 | 77 | 44 | 198 | 198 | 100 |
| WY | 21 | 21 | 100 | 30 | 30 | 100 | 30 | 30 | 100 | 21 | | 0 | 0 | 30 | 0 | 0 | 30 | 30 | 100 |

*Abbreviations:* EHE, “Ending the HIV Epidemic”; QHP, Qualified Health Plan; DTG/ABC/3TC, dolutegravir/abacavir/lamivudine; BIC/FTC/TAF, bictegravir/emtricitabine/tenofovir alafenamide fumarate
