# Supplementary material for: Access to a novel first-line single-tablet HIV antiretroviral regimen in Affordable Care Act Marketplace plans, 2018–2020
Source: J Pharm Policy Pract. 2023 Apr 20;16:57. doi: 10.1186/s40545-023-00559-8 (PMC10116786; doi:10.1186/s40545-023-00559-8)
Supplement: Supplementary file 2 — Additional file 2. Cost Sharing Structure for QHPs Coverage of DTG/ABC/3TC by Census Region, EHE Jurisdiction, and State, 2018–2020. Cost Sharing Structure for QHP Coverage of BIC/FTC/TAF by Census Region, EHE Jurisdiction, and State, 2018–2020. [file 40545_2023_559_MOESM2_ESM.docx]

**Additional File 2a.** Cost Sharing Structure for QHPs Coverage of DTG/ABC/3TC by Census Region, EHE Jurisdiction, and State, 2018 – 2020

|  | **DTG/ABC/3TC** | | | | | | | | | | | |
| --- | --- | --- | --- | --- | --- | --- | --- | --- | --- | --- | --- | --- |
|  | **2018** | | | | **2019** | | | | **2020** | | | |
| Characteristics | copay | % | coinsurance | % | copay | % | coinsurance | % | copay | % | coinsurance | % |
| National | 12108 | 67% | 6052 | 33% | 10856 | 66% | 5657 | 34% | 11659 | 59% | 8016 | 41% |
| Regional |  |  |  |  |  |  |  |  |  |  |  |  |
| Northeast | 1927 | 74% | 694 | 26% | 2420 | 78% | 645 | 21% | 2551 | 76% | 789 | 24% |
| Midwest | 2453 | 53% | 2197 | 47% | 2265 | 54% | 1945 | 46% | 2398 | 45% | 2921 | 55% |
| South | 6001 | 72% | 2360 | 28% | 4409 | 70% | 1932 | 30% | 4793 | 62% | 2998 | 38% |
| West | 1727 | 68% | 801 | 32% | 1762 | 61% | 1135 | 39% | 1917 | 59% | 1308 | 41% |
| EHE Status |  |  |  |  |  |  |  |  |  |  |  |  |
| EHE | 3712 | 73% | 1323 | 26% | 2998 | 75% | 1008 | 25% | 3168 | 62% | 1956 | 38% |
| Non-EHE | 8396 | 64% | 4729 | 36% | 7858 | 63% | 4649 | 37% | 8491 | 58% | 6060 | 42% |
| State |  |  |  |  |  |  |  |  |  |  |  |  |
| AK | 0 | 0% | 15 | 100% | 0 | 0% | 15 | 100% | 13 | 50% | 13 | 50% |
| AL | 123 | 89% | 15 | 11% | 96 | 99% | 1 | 1% | 91 | 100% | 0 | 0% |
| AR | 155 | 72% | 59 | 28% | 120 | 72% | 47 | 28% | 98 | 52% | 91 | 48% |
| AZ | 39 | 61% | 25 | 39% | 57 | 53% | 50 | 47% | 90 | 44% | 115 | 56% |
| CA | 735 | 74% | 259 | 26% | 742 | 72% | 282 | 28% | 847 | 78% | 240 | 22% |
| CO | 150 | 69% | 66 | 31% | 222 | 66% | 114 | 34% | 160 | 52% | 150 | 48% |
| CT | 120 | 47% | 136 | 53% | 32 | 14% | 192 | 86% | 24 | 9% | 248 | 91% |
| DC | 22 | 85% | 4 | 15% | 21 | 84% | 4 | 16% | 21 | 84% | 4 | 16% |
| DE | 3 | 43% | 4 | 57% | 2 | 25% | 6 | 75% | 8 | 73% | 3 | 27% |
| FL | 3291 | 84% | 640 | 16% | 1973 | 84% | 363 | 16% | 2286 | 79% | 595 | 21% |
| GA | 111 | 34% | 211 | 66% | 212 | 39% | 333 | 61% | 270 | 52% | 246 | 48% |
| HI | 23 | 52% | 21 | 48% | 24 | 53% | 21 | 47% | 24 | 53% | 21 | 47% |
| IA | 28 | 33% | 56 | 67% | 111 | 79% | 30 | 21% | 130 | 81% | 30 | 19% |
| ID | 242 | 77% | 73 | 23% | 206 | 65% | 113 | 35% | 183 | 69% | 83 | 31% |
| IL | 91 | 28% | 232 | 72% | 60 | 22% | 218 | 78% | 42 | 13% | 289 | 87% |
| IN | 265 | 60% | 175 | 40% | 256 | 68% | 119 | 32% | 256 | 48% | 272 | 52% |
| KS | 54 | 57% | 41 | 43% | 71 | 79% | 19 | 21% | 110 | 65% | 60 | 35% |
| KY | 78 | 55% | 64 | 45% | 72 | 54% | 61 | 46% | 72 | 54% | 61 | 46% |
| LA | 40 | 19% | 166 | 81% | 60 | 30% | 143 | 70% | 16 | 10% | 138 | 90% |
| MA | 526 | 89% | 65 | 11% | 321 | 98% | 7 | 2% | 436 | 98% | 7 | 2% |
| MD | 136 | 89% | 16 | 11% | 92 | 85% | 16 | 15% | 112 | 88% | 16 | 13% |
| ME | 8 | 9% | 86 | 91% | 34 | 15% | 193 | 85% | 30 | 11% | 254 | 89% |
| MI | 447 | 54% | 386 | 46% | 468 | 54% | 399 | 46% | 449 | 48% | 489 | 52% |
| MN | 99 | 27% | 267 | 73% | 150 | 39% | 239 | 61% | 221 | 44% | 280 | 56% |
| MO | 140 | 73% | 53 | 27% | 50 | 31% | 111 | 69% | 61 | 29% | 148 | 71% |
| MS | 90 | 52% | 84 | 48% | 12 | 40% | 18 | 60% | 6 | 11% | 48 | 89% |
| MT | 42 | 49% | 44 | 51% | 52 | 51% | 50 | 49% | 48 | 41% | 68 | 59% |
| NC | 45 | 27% | 119 | 73% | 74 | 39% | 118 | 61% | 96 | 45% | 115 | 55% |
| ND | 51 | 58% | 37 | 42% | 80 | 64% | 45 | 36% | 114 | 75% | 39 | 25% |
| NE | 14 | 33% | 28 | 67% | 35 | 69% | 16 | 31% | 52 | 74% | 18 | 26% |
| NH | 14 | 40% | 21 | 60% | 9 | 33% | 18 | 67% | 5 | 19% | 22 | 81% |
| NJ | 30 | 19% | 125 | 81% | 4 | 14% | 24 | 86% | 3 | 10% | 26 | 90% |
| NM | 98 | 58% | 70 | 42% | 120 | 59% | 85 | 41% | 85 | 44% | 110 | 56% |
| NV | 189 | 100% | 0 | 0% | 30 | 79% | 8 | 21% | 144 | 82% | 32 | 18% |
| NY | 1075 | 93% | 78 | 7% | 1769 | 95% | 86 | 5% | 1735 | 95% | 82 | 5% |
| OH | 411 | 51% | 396 | 49% | 466 | 52% | 422 | 48% | 462 | 35% | 843 | 65% |
| OK | 17 | 36% | 30 | 64% | 46 | 43% | 61 | 57% | 67 | 50% | 66 | 50% |
| OR | 136 | 74% | 47 | 26% | 152 | 78% | 42 | 22% | 180 | 65% | 99 | 35% |
| PA | 102 | 38% | 167 | 62% | 181 | 62% | 111 | 38% | 239 | 64% | 133 | 36% |
| RI | 28 | 100% | 0 | 0% | 52 | 100% | 0 | 0% | 56 | 100% | 0 | 0% |
| SC | 1380 | 79% | 368 | 21% | 1069 | 96% | 48 | 4% | 996 | 69% | 444 | 31% |
| SD | 64 | 63% | 38 | 37% | 42 | 51% | 40 | 49% | 42 | 51% | 40 | 49% |
| TN | 20 | 45% | 24 | 55% | 44 | 48% | 48 | 52% | 74 | 40% | 113 | 60% |
| TX | 294 | 51% | 282 | 49% | 300 | 43% | 398 | 57% | 339 | 29% | 827 | 71% |
| UT | 13 | 18% | 58 | 82% | 74 | 33% | 151 | 67% | 10 | 7% | 125 | 93% |
| VA | 113 | 44% | 141 | 56% | 122 | 40% | 185 | 60% | 95 | 35% | 179 | 65% |
| VT | 24 | 60% | 16 | 40% | 18 | 56% | 14 | 44% | 23 | 58% | 17 | 43% |
| WA | 45 | 28% | 117 | 72% | 59 | 23% | 198 | 77% | 109 | 31% | 246 | 69% |
| WI | 789 | 62% | 488 | 38% | 476 | 62% | 287 | 38% | 459 | 53% | 413 | 47% |
| WV | 83 | 38% | 133 | 62% | 94 | 53% | 82 | 47% | 146 | 74% | 52 | 26% |
| WY | 15 | 71% | 6 | 29% | 24 | 80% | 6 | 20% | 24 | 80% | 6 | 20% |

*Abbreviations:* EHE, “Ending the HIV Epidemic”; QHP, Qualified Health Plan; DTG/ABC/3TC, dolutegravir/abacavir/lamivudine *Footnote:* All QHPs providing coverage were included in % calculations, with exclusion of QHPs not providing coverage.

**Additional File 2b.** Cost Sharing Structure for QHP Coverage of BIC/FTC/TAF by Census Region, EHE Jurisdiction, and State, 2018 – 2020

|  | **BIC/FTC/TAF** | | | | | | | | | | | |
| --- | --- | --- | --- | --- | --- | --- | --- | --- | --- | --- | --- | --- |
|  | **2018** | | | | **2019** | | | | **2020** | | | |
| Characteristics | copay | % | coinsurance | % | copay | % | coinsurance | % | copay | % | coinsurance | % |
| National | 8075 | 69% | 3549 | 30% | 6761 | 68% | 3187 | 32% | 11229 | 61% | 7330 | 39% |
| Regional |  |  |  |  |  |  |  |  |  |  |  |  |
| Northeast | 1355 | 71% | 557 | 29% | 1696 | 80% | 417 | 20% | 2404 | 75% | 796 | 25% |
| Midwest | 445 | 36% | 775 | 64% | 625 | 47% | 698 | 53% | 2374 | 49% | 2516 | 51% |
| South | 4965 | 75% | 1631 | 25% | 3331 | 71% | 1389 | 29% | 4421 | 61% | 2824 | 39% |
| West | 1310 | 69% | 586 | 31% | 1109 | 62% | 683 | 38% | 2030 | 63% | 1194 | 37% |
| EHE Status |  |  |  |  |  |  |  |  |  |  |  |  |
| EHE | 2826 | 73% | 1000 | 26% | 2136 | 78% | 585 | 21% | 3069 | 62% | 1861 | 38% |
| Non-EHE | 5249 | 67% | 2549 | 33% | 4625 | 64% | 2602 | 36% | 8160 | 60% | 5469 | 40% |
| State |  |  |  |  |  |  |  |  |  |  |  |  |
| AK | 6 | 40% | 9 | 60% | 6 | 40% | 9 | 60% | 13 | 50% | 13 | 50% |
| AL | 117 | 90% | 13 | 10% | 0 | 0% | 0 | 0% | 91 | 100% | 0 | 0% |
| AR | 126 | 78% | 35 | 22% | 91 | 76% | 28 | 24% | 98 | 52% | 91 | 48% |
| AZ | 30 | 55% | 25 | 45% | 22 | 33% | 44 | 67% | 68 | 39% | 108 | 61% |
| CA | 403 | 79% | 108 | 21% | 413 | 76% | 132 | 24% | 843 | 78% | 232 | 22% |
| CO | 390 | 65% | 212 | 35% | 319 | 67% | 154 | 33% | 432 | 70% | 185 | 30% |
| CT | 104 | 41% | 152 | 59% | 80 | 45% | 96 | 55% | 24 | 9% | 248 | 91% |
| DC | 16 | 62% | 10 | 38% | 16 | 64% | 9 | 36% | 21 | 84% | 4 | 16% |
| DE | 3 | 43% | 4 | 57% | 2 | 25% | 6 | 75% | 8 | 73% | 3 | 27% |
| FL | 2866 | 76% | 891 | 24% | 1658 | 76% | 538 | 24% | 2028 | 70% | 853 | 30% |
| GA | 63 | 67% | 31 | 33% | 86 | 47% | 98 | 53% | 270 | 52% | 246 | 48% |
| HI | 0 | 0% | 0 | 0% | 4 | 36% | 7 | 64% | 12 | 36% | 21 | 64% |
| IA | 0 | 0% | 0 | 0% | 0 | 0% | 0 | 0% | 130 | 81% | 30 | 19% |
| ID | 221 | 86% | 36 | 14% | 182 | 71% | 75 | 29% | 171 | 74% | 60 | 26% |
| IL | 37 | 26% | 103 | 74% | 34 | 21% | 129 | 79% | 95 | 30% | 221 | 70% |
| IN | 0 | 0% | 0 | 0% | 0 | 0% | 0 | 0% | 256 | 48% | 272 | 52% |
| KS | 3 | 75% | 1 | 25% | 37 | 93% | 3 | 8% | 110 | 65% | 60 | 35% |
| KY | 0 | 0% | 0 | 0% | 0 | 0% | 0 | 0% | 72 | 54% | 61 | 46% |
| LA | 0 | 0% | 0 | 0% | 0 | 0% | 103 | 100% | 8 | 5% | 146 | 95% |
| MA | 470 | 91% | 49 | 9% | 316 | 98% | 7 | 2% | 436 | 98% | 7 | 2% |
| MD | 104 | 68% | 48 | 32% | 68 | 63% | 40 | 37% | 112 | 88% | 16 | 13% |
| ME | 8 | 9% | 86 | 91% | 9 | 5% | 168 | 95% | 30 | 11% | 254 | 89% |
| MI | 116 | 37% | 196 | 63% | 224 | 51% | 213 | 49% | 459 | 54% | 392 | 46% |
| MN | 19 | 19% | 83 | 81% | 6 | 9% | 60 | 91% | 168 | 47% | 186 | 53% |
| MO | 68 | 39% | 105 | 61% | 25 | 40% | 37 | 60% | 54 | 29% | 135 | 71% |
| MS | 90 | 52% | 84 | 48% | 12 | 40% | 18 | 60% | 6 | 11% | 48 | 89% |
| MT | 4 | 100% | 0 | 0% | 0 | 0% | 0 | 0% | 12 | 30% | 28 | 70% |
| NC | 0 | 0% | 18 | 100% | 74 | 39% | 118 | 61% | 84 | 43% | 112 | 57% |
| ND | 20 | 56% | 16 | 44% | 16 | 57% | 12 | 43% | 114 | 75% | 39 | 25% |
| NE | 0 | 0% | 0 | 0% | 0 | 0% | 4 | 100% | 52 | 74% | 18 | 26% |
| NH | 8 | 33% | 16 | 67% | 5 | 29% | 12 | 71% | 5 | 19% | 22 | 81% |
| NJ | 12 | 11% | 95 | 89% | 2 | 10% | 19 | 90% | 3 | 10% | 26 | 90% |
| NM | 20 | 31% | 45 | 69% | 30 | 43% | 40 | 57% | 50 | 48% | 55 | 52% |
| NV | 102 | 95% | 5 | 5% | 12 | 60% | 8 | 40% | 121 | 69% | 55 | 31% |
| NY | 626 | 97% | 20 | 3% | 1109 | 98% | 24 | 2% | 1579 | 94% | 98 | 6% |
| OH | 54 | 43% | 71 | 57% | 106 | 57% | 79 | 43% | 477 | 40% | 719 | 60% |
| OK | 5 | 100% | 0 | 0% | 10 | 18% | 45 | 82% | 67 | 50% | 66 | 50% |
| OR | 70 | 69% | 31 | 31% | 39 | 54% | 33 | 46% | 152 | 63% | 91 | 37% |
| PA | 81 | 38% | 132 | 62% | 132 | 61% | 86 | 39% | 239 | 64% | 133 | 36% |
| RI | 27 | 100% | 0 | 0% | 30 | 100% | 0 | 0% | 56 | 100% | 0 | 0% |
| SC | 1380 | 79% | 368 | 21% | 1069 | 96% | 48 | 4% | 996 | 69% | 444 | 31% |
| SD | 18 | 100% | 0 | 0% | 0 | 0% | 0 | 0% | 42 | 51% | 40 | 49% |
| TN | 0 | 0% | 0 | 0% | 6 | 60% | 4 | 40% | 28 | 42% | 38 | 58% |
| TX | 143 | 63% | 85 | 37% | 134 | 43% | 177 | 57% | 297 | 38% | 479 | 62% |
| UT | 6 | 23% | 20 | 77% | 0 | 0% | 20 | 100% | 35 | 26% | 100 | 74% |
| VA | 52 | 54% | 44 | 46% | 83 | 45% | 102 | 55% | 89 | 35% | 165 | 65% |
| VT | 19 | 73% | 7 | 27% | 13 | 72% | 5 | 28% | 32 | 80% | 8 | 20% |
| WA | 58 | 38% | 95 | 62% | 82 | 34% | 161 | 66% | 97 | 29% | 240 | 71% |
| WI | 110 | 35% | 200 | 65% | 177 | 52% | 161 | 48% | 417 | 51% | 404 | 49% |
| WV | 0 | 0% | 0 | 0% | 22 | 29% | 55 | 71% | 146 | 74% | 52 | 26% |
| WY | 0 | 0% | 0 | 0% | 0 | 0% | 0 | 0% | 24 | 80% | 6 | 20% |

*Abbreviations:* EHE, “Ending the HIV Epidemic”; QHP, Qualified Health Plan; BIC/FTC/TAF, bictegravir/emtricitabine/tenofovir alafenamide fumarate *Footnote:* All QHPs providing coverage were included in % calculations, with exclusion of QHPs not providing coverage.
