# Supplementary material for: Access to a novel first-line single-tablet HIV antiretroviral regimen in Affordable Care Act Marketplace plans, 2018–2020
Source: J Pharm Policy Pract. 2023 Apr 20;16:57. doi: 10.1186/s40545-023-00559-8 (PMC10116786; doi:10.1186/s40545-023-00559-8)
Supplement: Supplementary file 3 — Additional file 3. Drug Tiering for QHP Coverage of DTG/ABC/3TC by Census Region, EHE Jurisdiction, and State, 2018–2020. Drug Tiering for QHP Coverage of BIC/FTC/TAF by Census Region, EHE Jurisdiction, and State, 2018–2020. [file 40545_2023_559_MOESM3_ESM.docx]

**Additional File 3a:** Drug Tiering for QHP Coverage of DTG/ABC/3TC by Census Region, EHE Jurisdiction, and State, 2018 – 2020

|  | **DTG/ABC/3TC** | | | | | | | | | | | | | |
| --- | --- | --- | --- | --- | --- | --- | --- | --- | --- | --- | --- | --- | --- | --- |
|  | **2018** | | | | **2019** | | | | | **2020** | | | | |
| Characteristics | Specialty | % | Non-specialty | % | | Specialty | % | Non-specialty | % | | Specialty | % | Non-specialty | % |
| National | 2509 | 14% | 15651 | 86% | | 2950 | 18% | 13563 | 82% | | 3746 | 19% | 12767 | 77% |
| Regional |  |  |  |  | |  |  |  |  | |  |  |  |  |
| Northeast | 194 | 7% | 2427 | 93% | | 326 | 11% | 2739 | 89% | | 614 | 18% | 2451 | 80% |
| Midwest | 1375 | 30% | 3275 | 70% | | 1315 | 31% | 2895 | 69% | | 1780 | 33% | 2430 | 58% |
| South | 692 | 8% | 7669 | 92% | | 833 | 13% | 5508 | 87% | | 748 | 10% | 5593 | 88% |
| West | 248 | 10% | 2280 | 90% | | 476 | 16% | 2421 | 84% | | 604 | 19% | 2293 | 79% |
| EHE Status |  |  |  |  | |  |  |  |  | |  |  |  |  |
| EHE | 319 | 6% | 4716 | 94% | | 382 | 10% | 3624 | 90% | | 471 | 9% | 3535 | 88% |
| Non-EHE | 2190 | 17% | 10935 | 83% | | 2568 | 21% | 9939 | 79% | | 3275 | 23% | 9232 | 74% |
| State |  |  |  |  | |  |  |  |  | |  |  |  |  |
| AK | 0 | 0% | 15 | 100% | | 0 | 0% | 15 | 100% | | 0 | 0% | 26 | 100% |
| AL | 0 | 0% | 138 | 100% | | 0 | 0% | 97 | 100% | | 0 | 0% | 91 | 100% |
| AR | 0 | 0% | 214 | 100% | | 0 | 0% | 167 | 100% | | 0 | 0% | 189 | 100% |
| AZ | 1 | 2% | 63 | 98% | | 0 | 0% | 107 | 100% | | 0 | 0% | 205 | 100% |
| CA | 35 | 4% | 959 | 96% | | 33 | 3% | 991 | 97% | | 35 | 3% | 1052 | 97% |
| CO | 6 | 3% | 210 | 97% | | 195 | 58% | 141 | 42% | | 282 | 91% | 28 | 9% |
| CT | 0 | 0% | 256 | 100% | | 112 | 50% | 112 | 50% | | 272 | 100% | 0 | 0% |
| DC | 0 | 0% | 26 | 100% | | 0 | 0% | 25 | 100% | | 0 | 0% | 25 | 100% |
| DE | 0 | 0% | 7 | 100% | | 0 | 0% | 8 | 100% | | 0 | 0% | 11 | 100% |
| FL | 0 | 0% | 3931 | 100% | | 0 | 0% | 2336 | 100% | | 0 | 0% | 2881 | 100% |
| GA | 228 | 71% | 94 | 29% | | 361 | 66% | 184 | 34% | | 206 | 40% | 310 | 60% |
| HI | 0 | 0% | 44 | 100% | | 0 | 0% | 45 | 100% | | 0 | 0% | 45 | 100% |
| IA | 0 | 0% | 84 | 100% | | 0 | 0% | 141 | 100% | | 0 | 0% | 160 | 100% |
| ID | 42 | 13% | 273 | 87% | | 42 | 13% | 277 | 87% | | 42 | 16% | 224 | 84% |
| IL | 183 | 57% | 140 | 43% | | 112 | 40% | 166 | 60% | | 148 | 45% | 183 | 55% |
| IN | 0 | 0% | 440 | 100% | | 0 | 0% | 375 | 100% | | 1 | 0% | 527 | 100% |
| KS | 0 | 0% | 95 | 100% | | 0 | 0% | 90 | 100% | | 0 | 0% | 170 | 100% |
| KY | 58 | 41% | 84 | 59% | | 61 | 46% | 72 | 54% | | 61 | 46% | 72 | 54% |
| LA | 46 | 22% | 160 | 78% | | 0 | 0% | 203 | 100% | | 0 | 0% | 154 | 100% |
| MA | 0 | 0% | 591 | 100% | | 0 | 0% | 328 | 100% | | 0 | 0% | 443 | 100% |
| MD | 0 | 0% | 152 | 100% | | 0 | 0% | 108 | 100% | | 0 | 0% | 128 | 100% |
| ME | 44 | 47% | 50 | 53% | | 110 | 48% | 117 | 52% | | 210 | 74% | 74 | 26% |
| MI | 260 | 31% | 573 | 69% | | 310 | 36% | 557 | 64% | | 367 | 39% | 571 | 61% |
| MN | 1 | 0% | 365 | 100% | | 3 | 1% | 386 | 99% | | 12 | 2% | 489 | 98% |
| MO | 27 | 14% | 166 | 86% | | 72 | 45% | 89 | 55% | | 90 | 43% | 119 | 57% |
| MS | 0 | 0% | 174 | 100% | | 0 | 0% | 30 | 100% | | 0 | 0% | 54 | 100% |
| MT | 38 | 44% | 48 | 56% | | 38 | 37% | 64 | 63% | | 40 | 34% | 76 | 66% |
| NC | 146 | 89% | 18 | 11% | | 165 | 86% | 27 | 14% | | 164 | 78% | 47 | 22% |
| ND | 0 | 0% | 88 | 100% | | 0 | 0% | 125 | 100% | | 0 | 0% | 153 | 100% |
| NE | 0 | 0% | 42 | 100% | | 0 | 0% | 51 | 100% | | 0 | 0% | 70 | 100% |
| NH | 11 | 31% | 24 | 69% | | 10 | 37% | 17 | 63% | | 20 | 74% | 7 | 26% |
| NJ | 1 | 1% | 154 | 99% | | 0 | 0% | 28 | 100% | | 0 | 0% | 29 | 100% |
| NM | 55 | 33% | 113 | 67% | | 55 | 27% | 150 | 73% | | 30 | 15% | 165 | 85% |
| NV | 0 | 0% | 189 | 100% | | 0 | 0% | 38 | 100% | | 44 | 25% | 132 | 75% |
| NY | 0 | 0% | 1153 | 100% | | 0 | 0% | 1855 | 100% | | 24 | 1% | 1793 | 99% |
| OH | 275 | 34% | 532 | 66% | | 437 | 49% | 451 | 51% | | 633 | 49% | 672 | 51% |
| OK | 0 | 0% | 47 | 100% | | 0 | 0% | 107 | 100% | | 0 | 0% | 133 | 100% |
| OR | 0 | 0% | 183 | 100% | | 0 | 0% | 194 | 100% | | 57 | 20% | 222 | 80% |
| PA | 138 | 51% | 131 | 49% | | 94 | 32% | 198 | 68% | | 88 | 24% | 284 | 76% |
| RI | 0 | 0% | 28 | 100% | | 0 | 0% | 52 | 100% | | 0 | 0% | 56 | 100% |
| SC | 0 | 0% | 1748 | 100% | | 0 | 0% | 1117 | 100% | | 0 | 0% | 1440 | 100% |
| SD | 32 | 31% | 70 | 69% | | 34 | 41% | 48 | 59% | | 34 | 41% | 48 | 59% |
| TN | 24 | 55% | 20 | 45% | | 24 | 26% | 68 | 74% | | 58 | 31% | 129 | 69% |
| TX | 72 | 13% | 504 | 88% | | 58 | 8% | 640 | 92% | | 84 | 7% | 1082 | 93% |
| UT | 35 | 49% | 36 | 51% | | 60 | 27% | 165 | 73% | | 70 | 52% | 65 | 48% |
| VA | 118 | 46% | 136 | 54% | | 164 | 53% | 143 | 47% | | 175 | 64% | 99 | 36% |
| VT | 0 | 0% | 40 | 100% | | 0 | 0% | 32 | 100% | | 0 | 0% | 40 | 100% |
| WA | 36 | 22% | 126 | 78% | | 53 | 21% | 204 | 79% | | 4 | 1% | 351 | 99% |
| WI | 597 | 47% | 680 | 53% | | 347 | 45% | 416 | 55% | | 495 | 57% | 377 | 43% |
| WV | 0 | 0% | 216 | 100% | | 0 | 0% | 176 | 100% | | 0 | 0% | 198 | 100% |
| WY | 0 | 0% | 21 | 100% | | 0 | 0% | 30 | 100% | | 0 | 0% | 30 | 100% |

*Abbreviations:* EHE, “Ending the HIV Epidemic”; QHP, Qualified Health Plan; DTG/ABC/3TC, dolutegravir/abacavir/lamivudine; BIC/FTC/TAF, bictegravir/emtricitabine/tenofovir alafenamide fumarate *Footnote:* All QHPs providing coverage were included in % calculations, with exclusion of QHPs not providing coverage.

**Additional File 3b:** Drug Tiering for QHP Coverage of BIC/FTC/TAF by Census Region, EHE Jurisdiction, and State, 2018 – 2020

|  | **BIC/FTC/TAF** | | | | | | | | | | | | |
| --- | --- | --- | --- | --- | --- | --- | --- | --- | --- | --- | --- | --- | --- |
|  | **2018** | | | | | **2019** | | | | **2020** | | | |
| Characteristics | Specialty | % | Non-specialty | % | Specialty | | % | Non-specialty | % | Specialty | % | Non-specialty | % |
| National | 1017 | 9% | 10607 | 91% | 1289 | | 13% | 10335 | 89% | 3613 | 19% | 14946 | 81% |
| Regional |  |  |  |  |  | |  |  |  |  |  |  |  |
| Northeast | 218 | 11% | 1694 | 89% | 289 | | 14% | 1623 | 85% | 614 | 19% | 2586 | 81% |
| Midwest | 498 | 41% | 722 | 59% | 558 | | 42% | 662 | 54% | 1647 | 34% | 3243 | 66% |
| South | 214 | 3% | 6382 | 97% | 329 | | 7% | 6267 | 95% | 821 | 11% | 6424 | 89% |
| West | 87 | 5% | 1809 | 95% | 113 | | 6% | 1783 | 94% | 531 | 16% | 2693 | 84% |
| EHE Status |  |  |  |  |  | |  |  |  |  |  |  |  |
| EHE | 85 | 2% | 3741 | 98% | 108 | | 4% | 3718 | 97% | 461 | 9% | 4469 | 91% |
| Non-EHE | 932 | 12% | 6866 | 88% | 1181 | | 16% | 6617 | 85% | 3152 | 23% | 10477 | 77% |
| State |  |  |  |  |  | |  |  |  |  |  |  |  |
| AK | 0 | 0% | 15 | 100% | 0 | | 0% | 15 | 100% | 0 | 0% | 26 | 100% |
| AL | 0 | 0% | 130 | 100% | 0 | | 0% | 0 | 0% | 0 | 0% | 91 | 100% |
| AR | 0 | 0% | 161 | 100% | 0 | | 0% | 119 | 100% | 0 | 0% | 189 | 100% |
| AZ | 0 | 0% | 55 | 100% | 0 | | 0% | 66 | 100% | 0 | 0% | 176 | 100% |
| CA | 0 | 0% | 511 | 100% | 9 | | 2% | 536 | 98% | 23 | 2% | 1052 | 98% |
| CO | 0 | 0% | 602 | 100% | 0 | | 0% | 473 | 100% | 201 | 33% | 416 | 67% |
| CT | 0 | 0% | 256 | 100% | 64 | | 36% | 112 | 64% | 272 | 100% | 0 | 0% |
| DC | 0 | 0% | 26 | 100% | 0 | | 0% | 25 | 100% | 0 | 0% | 25 | 100% |
| DE | 0 | 0% | 7 | 100% | 0 | | 0% | 8 | 100% | 0 | 0% | 11 | 100% |
| FL | 170 | 5% | 3587 | 95% | 92 | | 4% | 2104 | 96% | 88 | 3% | 2793 | 97% |
| GA | 18 | 19% | 76 | 81% | 16 | | 9% | 168 | 91% | 206 | 40% | 310 | 60% |
| HI | 0 | 0% | 0 | 0% | 0 | | 0% | 11 | 100% | 0 | 0% | 33 | 100% |
| IA | 0 | 0% | 0 | 0% | 0 | | 0% | 0 | 0% | 0 | 0% | 160 | 100% |
| ID | 0 | 0% | 257 | 100% | 0 | | 0% | 257 | 100% | 42 | 18% | 189 | 82% |
| IL | 0 | 0% | 140 | 100% | 12 | | 7% | 151 | 93% | 52 | 16% | 264 | 84% |
| IN | 0 | 0% | 0 | 0% | 0 | | 0% | 0 | 0% | 1 | 0% | 527 | 100% |
| KS | 0 | 0% | 4 | 100% | 0 | | 0% | 40 | 100% | 0 | 0% | 170 | 100% |
| KY | 0 | 0% | 0 | 0% | 0 | | 0% | 0 | 0% | 61 | 46% | 72 | 54% |
| LA | 0 | 0% | 0 | 0% | 0 | | 0% | 103 | 100% | 48 | 31% | 106 | 69% |
| MA | 0 | 0% | 519 | 100% | 0 | | 0% | 323 | 100% | 0 | 0% | 443 | 100% |
| MD | 0 | 0% | 152 | 100% | 0 | | 0% | 108 | 100% | 0 | 0% | 128 | 100% |
| ME | 70 | 74% | 24 | 26% | 127 | | 72% | 50 | 28% | 210 | 74% | 74 | 26% |
| MI | 90 | 29% | 222 | 71% | 164 | | 38% | 273 | 62% | 280 | 33% | 571 | 67% |
| MN | 1 | 1% | 101 | 99% | 3 | | 5% | 63 | 95% | 12 | 3% | 342 | 97% |
| MO | 0 | 0% | 173 | 100% | 0 | | 0% | 62 | 100% | 85 | 45% | 104 | 55% |
| MS | 0 | 0% | 174 | 100% | 0 | | 0% | 30 | 100% | 0 | 0% | 54 | 100% |
| MT | 0 | 0% | 4 | 100% | 0 | | 0% | 0 | 0% | 40 | 100% | 0 | 0% |
| NC | 0 | 0% | 18 | 100% | 165 | | 86% | 27 | 14% | 164 | 84% | 32 | 16% |
| ND | 0 | 0% | 36 | 100% | 0 | | 0% | 28 | 100% | 0 | 0% | 153 | 100% |
| NE | 0 | 0% | 0 | 0% | 0 | | 0% | 4 | 100% | 0 | 0% | 70 | 100% |
| NH | 10 | 42% | 14 | 58% | 4 | | 24% | 13 | 76% | 20 | 74% | 7 | 26% |
| NJ | 0 | 0% | 107 | 100% | 0 | | 0% | 21 | 100% | 0 | 0% | 29 | 100% |
| NM | 55 | 85% | 10 | 15% | 55 | | 79% | 15 | 21% | 30 | 29% | 75 | 71% |
| NV | 0 | 0% | 107 | 100% | 0 | | 0% | 20 | 100% | 64 | 36% | 112 | 64% |
| NY | 0 | 0% | 646 | 100% | 0 | | 0% | 1133 | 100% | 24 | 1% | 1653 | 99% |
| OH | 97 | 78% | 28 | 22% | 132 | | 71% | 53 | 29% | 633 | 53% | 563 | 47% |
| OK | 0 | 0% | 5 | 100% | 0 | | 0% | 55 | 100% | 0 | 0% | 133 | 100% |
| OR | 0 | 0% | 101 | 100% | 0 | | 0% | 72 | 100% | 57 | 23% | 186 | 77% |
| PA | 138 | 65% | 75 | 35% | 94 | | 43% | 124 | 57% | 88 | 24% | 284 | 76% |
| RI | 0 | 0% | 27 | 100% | 0 | | 0% | 30 | 100% | 0 | 0% | 56 | 100% |
| SC | 0 | 0% | 1748 | 100% | 0 | | 0% | 1117 | 100% | 0 | 0% | 1440 | 100% |
| SD | 0 | 0% | 18 | 100% | 0 | | 0% | 0 | 0% | 34 | 41% | 48 | 59% |
| TN | 0 | 0% | 0 | 0% | 0 | | 0% | 10 | 100% | 0 | 0% | 66 | 100% |
| TX | 16 | 7% | 212 | 93% | 0 | | 0% | 311 | 100% | 84 | 11% | 692 | 89% |
| UT | 0 | 0% | 26 | 100% | 0 | | 0% | 20 | 100% | 70 | 52% | 65 | 48% |
| VA | 10 | 10% | 86 | 90% | 56 | | 30% | 129 | 70% | 170 | 67% | 84 | 33% |
| VT | 0 | 0% | 26 | 100% | 0 | | 0% | 18 | 100% | 0 | 0% | 40 | 100% |
| WA | 32 | 21% | 121 | 79% | 49 | | 20% | 194 | 80% | 4 | 1% | 333 | 99% |
| WI | 310 | 100% | 0 | 0% | 247 | | 73% | 91 | 27% | 550 | 67% | 271 | 33% |
| WV | 0 | 0% | 0 | 0% | 0 | | 0% | 77 | 100% | 0 | 0% | 198 | 100% |
| WY | 0 | 0% | 0 | 0% | 0 | | 0% | 0 | 0% | 0 | 0% | 30 | 100% |

*Abbreviations:* EHE, “Ending the HIV Epidemic”; QHP, Qualified Health Plan; BIC/FTC/TAF, bictegravir/emtricitabine/tenofovir alafenamide fumarate *Footnote:* All QHPs providing coverage were included in % calculations, with exclusion of QHPs not providing coverage.
