# Supplementary material for: Access to a novel first-line single-tablet HIV antiretroviral regimen in Affordable Care Act Marketplace plans, 2018–2020
Source: J Pharm Policy Pract. 2023 Apr 20;16:57. doi: 10.1186/s40545-023-00559-8 (PMC10116786; doi:10.1186/s40545-023-00559-8)
Supplement: Supplementary file 4 — Additional file 4. Prior Authorization Requirement for DTG/ABC/3TC and BIC/FTC/TAF by Census Region, EHE Jurisdiction, and State, 2018–2020. [file 40545_2023_559_MOESM4_ESM.docx]

**Additional File 4.** Prior Authorization Requirement for DTG/ABC/3TC and BIC/FTC/TAF by Census Region, EHE Jurisdiction, and State, 2018 – 2020

|  | **DTG/ABC/3TC** | | | | | | | | **BIC/FTC/TAF** | | | | | | | |
| --- | --- | --- | --- | --- | --- | --- | --- | --- | --- | --- | --- | --- | --- | --- | --- | --- |
|  | **2018** | | | **2019** | | | **2020** | | **2018** | | | **2019** | | | **2020** | |
| Characteristics | *n* | % | *n* | | % | *n* | | % | *n* | % | *n* | | % | *n* | | % |
| National | 294 | 2% | 289 | | 2% | 106 | | 1% | 589 | 5% | 836 | | 8% | 0 | | 0% |
| Regional |  |  |  | |  |  | |  |  |  |  | |  |  | |  |
| Northeast | 111 | 4% | 111 | | 4% | 35 | | 1% | 357 | 19% | 717 | | 34% | 0 | | 0% |
| Midwest | 183 | 4% | 178 | | 4% | 71 | | 1% | 16 | 1% | 0 | | 0% | 0 | | 0% |
| South | 0 | 0% | 0 | | 0% | 0 | | 0% | 184 | 3% | 0 | | 0% | 0 | | 0% |
| West | 0 | 0% | 0 | | 0% | 0 | | 0% | 32 | 2% | 119 | | 7% | 0 | | 0% |
| EHE Status |  |  |  | |  |  | |  |  |  |  | |  |  | |  |
| EHE | 52 | 1% | 27 | | 1% | 11 | | 0% | 257 | 7% | 136 | | 5% | 0 | | 0% |
| Non-EHE | 242 | 2% | 262 | | 2% | 95 | | 1% | 332 | 4% | 700 | | 10% | 0 | | 0% |
| State |  |  |  | |  |  | |  |  |  |  | |  |  | |  |
| AK | 0 | 0% | 0 | | 0% | 0 | | 0% | 0 | 0% | 0 | | 0% | 0 | | 0% |
| AL | 0 | 0% | 0 | | 0% | 0 | | 0% | 0 | 0% | 0 | | 0% | 0 | | 0% |
| AR | 0 | 0% | 0 | | 0% | 0 | | 0% | 0 | 0% | 0 | | 0% | 0 | | 0% |
| AZ | 0 | 0% | 0 | | 0% | 0 | | 0% | 0 | 0% | 0 | | 0% | 0 | | 0% |
| CA | 0 | 0% | 0 | | 0% | 0 | | 0% | 0 | 0% | 0 | | 0% | 0 | | 0% |
| CO | 0 | 0% | 0 | | 0% | 0 | | 0% | 0 | 0% | 0 | | 0% | 0 | | 0% |
| CT | 0 | 0% | 0 | | 0% | 0 | | 0% | 0 | 0% | 0 | | 0% | 0 | | 0% |
| DC | 0 | 0% | 0 | | 0% | 0 | | 0% | 0 | 0% | 0 | | 0% | 0 | | 0% |
| DE | 0 | 0% | 0 | | 0% | 0 | | 0% | 0 | 0% | 0 | | 0% | 0 | | 0% |
| FL | 0 | 0% | 0 | | 0% | 0 | | 0% | 0 | 0% | 0 | | 0% | 0 | | 0% |
| GA | 0 | 0% | 0 | | 0% | 0 | | 0% | 0 | 0% | 0 | | 0% | 0 | | 0% |
| HI | 0 | 0% | 0 | | 0% | 0 | | 0% | 0 | 0% | 0 | | 0% | 0 | | 0% |
| IA | 0 | 0% | 0 | | 0% | 0 | | 0% | 0 | 0% | 0 | | 0% | 0 | | 0% |
| ID | 0 | 0% | 0 | | 0% | 0 | | 0% | 0 | 0% | 70 | | 27% | 0 | | 0% |
| IL | 0 | 0% | 0 | | 0% | 0 | | 0% | 0 | 0% | 0 | | 0% | 0 | | 0% |
| IN | 0 | 0% | 0 | | 0% | 0 | | 0% | 0 | 0% | 0 | | 0% | 0 | | 0% |
| KS | 0 | 0% | 0 | | 0% | 0 | | 0% | 4 | 100% | 0 | | 0% | 0 | | 0% |
| KY | 0 | 0% | 0 | | 0% | 0 | | 0% | 0 | 0% | 0 | | 0% | 0 | | 0% |
| LA | 0 | 0% | 0 | | 0% | 0 | | 0% | 0 | 0% | 0 | | 0% | 0 | | 0% |
| MA | 0 | 0% | 0 | | 0% | 0 | | 0% | 0 | 0% | 0 | | 0% | 0 | | 0% |
| MD | 0 | 0% | 0 | | 0% | 0 | | 0% | 32 | 21% | 0 | | 0% | 0 | | 0% |
| ME | 0 | 0% | 0 | | 0% | 0 | | 0% | 0 | 0% | 0 | | 0% | 0 | | 0% |
| MI | 156 | 19% | 178 | | 21% | 66 | | 7% | 0 | 0% | 0 | | 0% | 0 | | 0% |
| MN | 0 | 0% | 0 | | 0% | 0 | | 0% | 0 | 0% | 0 | | 0% | 0 | | 0% |
| MO | 27 | 14% | 0 | | 0% | 5 | | 2% | 0 | 0% | 0 | | 0% | 0 | | 0% |
| MS | 0 | 0% | 0 | | 0% | 0 | | 0% | 144 | 83% | 0 | | 0% | 0 | | 0% |
| MT | 0 | 0% | 0 | | 0% | 0 | | 0% | 0 | 0% | 0 | | 0% | 0 | | 0% |
| NC | 0 | 0% | 0 | | 0% | 0 | | 0% | 0 | 0% | 0 | | 0% | 0 | | 0% |
| ND | 0 | 0% | 0 | | 0% | 0 | | 0% | 0 | 0% | 0 | | 0% | 0 | | 0% |
| NE | 0 | 0% | 0 | | 0% | 0 | | 0% | 0 | 0% | 0 | | 0% | 0 | | 0% |
| NH | 0 | 0% | 0 | | 0% | 0 | | 0% | 0 | 0% | 0 | | 0% | 0 | | 0% |
| NJ | 0 | 0% | 0 | | 0% | 0 | | 0% | 0 | 0% | 0 | | 0% | 0 | | 0% |
| NM | 0 | 0% | 0 | | 0% | 0 | | 0% | 0 | 0% | 0 | | 0% | 0 | | 0% |
| NV | 0 | 0% | 0 | | 0% | 0 | | 0% | 0 | 0% | 0 | | 0% | 0 | | 0% |
| NY | 111 | 10% | 111 | | 6% | 35 | | 2% | 357 | 55% | 705 | | 62% | 0 | | 0% |
| OH | 0 | 0% | 0 | | 0% | 0 | | 0% | 12 | 10% | 0 | | 0% | 0 | | 0% |
| OK | 0 | 0% | 0 | | 0% | 0 | | 0% | 0 | 0% | 0 | | 0% | 0 | | 0% |
| OR | 0 | 0% | 0 | | 0% | 0 | | 0% | 0 | 0% | 0 | | 0% | 0 | | 0% |
| PA | 0 | 0% | 0 | | 0% | 0 | | 0% | 0 | 0% | 12 | | 6% | 0 | | 0% |
| RI | 0 | 0% | 0 | | 0% | 0 | | 0% | 0 | 0% | 0 | | 0% | 0 | | 0% |
| SC | 0 | 0% | 0 | | 0% | 0 | | 0% | 0 | 0% | 0 | | 0% | 0 | | 0% |
| SD | 0 | 0% | 0 | | 0% | 0 | | 0% | 0 | 0% | 0 | | 0% | 0 | | 0% |
| TN | 0 | 0% | 0 | | 0% | 0 | | 0% | 0 | 0% | 0 | | 0% | 0 | | 0% |
| TX | 0 | 0% | 0 | | 0% | 0 | | 0% | 6 | 3% | 0 | | 0% | 0 | | 0% |
| UT | 0 | 0% | 0 | | 0% | 0 | | 0% | 0 | 0% | 0 | | 0% | 0 | | 0% |
| VA | 0 | 0% | 0 | | 0% | 0 | | 0% | 2 | 2% | 0 | | 0% | 0 | | 0% |
| VT | 0 | 0% | 0 | | 0% | 0 | | 0% | 0 | 0% | 0 | | 0% | 0 | | 0% |
| WA | 0 | 0% | 0 | | 0% | 0 | | 0% | 32 | 21% | 49 | | 20% | 18 | | 5% |
| WI | 0 | 0% | 0 | | 0% | 0 | | 0% | 0 | 0% | 0 | | 0% | 0 | | 0% |
| WV | 0 | 0% | 0 | | 0% | 0 | | 0% | 0 | 0% | 0 | | 0% | 0 | | 0% |
| WY | 0 | 0% | 0 | | 0% | 0 | | 0% | 0 | 0% | 0 | | 0% | 0 | | 0% |

*Abbreviations:* EHE, “Ending the HIV Epidemic”; QHP, Qualified Health Plan; DTG/ABC/3TC, dolutegravir/abacavir/lamivudine; BIC/FTC/TAF, bictegravir/emtricitabine/tenofovir alafenamide fumarate *Footnote:* All QHPs providing coverage were included in % calculations, with exclusion of QHPs not providing coverage.
