# Supplementary material for: Access to a novel first-line single-tablet HIV antiretroviral regimen in Affordable Care Act Marketplace plans, 2018–2020
Source: J Pharm Policy Pract. 2023 Apr 20;16:57. doi: 10.1186/s40545-023-00559-8 (PMC10116786; doi:10.1186/s40545-023-00559-8)
Supplement: Supplementary file 5 — Additional file 5. Average Out-of-Pocket (OOP) Cost per Month for QHP Coverage of DTG/ABC/3TC and BIC/FTC/TAF by U.S., Census Region, EHE Jurisdiction, and State, 2018–2020. [file 40545_2023_559_MOESM5_ESM.docx]

**Additional File 5.** Average Out-of-Pocket (OOP) Cost per Month for QHP Coverage of DTG/ABC/3TC and BIC/FTC/TAF by U.S., Census Region, EHE Jurisdiction, and State, 2018 – 2020

|  | **DTG/ABC/3TC** | | | | | | **BIC/FTC/TAF** | | | | | |
| --- | --- | --- | --- | --- | --- | --- | --- | --- | --- | --- | --- | --- |
|  | **2018** | | **2019** | | **2020** | | **2018** | | **2019** | | **2020** | |
| Characteristics | Copay | Coinsurance | Copay | Coinsurance | Copay | Coinsurance | Copay | Coinsurance | Copay | Coinsurance | Copay | Coinsurance |
| National | $681 | $958 | $737 | $1,002 | $739 | $1,008 | $696 | $945 | $744 | $1,003 | $733 | $1,012 |
| Regional |  |  |  |  |  |  |  |  |  |  |  |  |
| Midwest | $693 | $952 | $705 | $975 | $728 | $995 | $826 | $990 | $732 | $1,001 | $688 | $1,005 |
| Northeast | $756 | $966 | $874 | $1,018 | $860 | $1,050 | $730 | $954 | $832 | $996 | $840 | $1,053 |
| West | $618 | $915 | $687 | $987 | $686 | $991 | $613 | $917 | $653 | $966 | $668 | $982 |
| South | $669 | $976 | $699 | $1,031 | $702 | $1,018 | $697 | $932 | $731 | $1,024 | $729 | $1,019 |
| EHE Status |  |  |  |  |  |  |  |  |  |  |  |  |
| EHE | $602 | $912 | $680 | $973 | $677 | $996 | $608 | $920 | $684 | $971 | $679 | $997 |
| Non-EHE | $716 | $971 | $760 | $1,008 | $762 | $1,012 | $743 | $956 | $771 | $1,010 | $753 | $1,017 |
| State |  |  |  |  |  |  |  |  |  |  |  |  |
| AK | -- | $1,104 | -- | $1,075 | $585 | $1,015 | $659 | $1,065 | $621 | $1,041 | $585 | $1,015 |
| AL | $693 | $848 | $681 | $927 | $700 | -- | $692 | $829 | -- | -- | $700 | -- |
| AR | $580 | $799 | $572 | $867 | $604 | $923 | $620 | $817 | $584 | $916 | $604 | $923 |
| AZ | $937 | $1,064 | $752 | $1,017 | $837 | $1,033 | $1,048 | $1,064 | $980 | $1,024 | $907 | $1,037 |
| CA | $571 | $842 | $610 | $901 | $645 | $961 | $586 | $866 | $617 | $921 | $640 | $960 |
| CO | $636 | $977 | $839 | $1,048 | $802 | $949 | $559 | $941 | $580 | $953 | $627 | $913 |
| CT | $600 | $897 | $806 | $1,012 | $901 | $1,058 | $697 | $957 | $755 | $997 | $901 | $1,060 |
| DC | $418 | $775 | $423 | $828 | $457 | $861 | $432 | $785 | $422 | $840 | $457 | $861 |
| DE | $955 | $946 | $1,002 | $1,145 | $750 | $965 | $955 | $961 | $1,002 | $1,145 | $750 | $965 |
| FL | $740 | $954 | $714 | $1,020 | $710 | $1,013 | $792 | $955 | $785 | $1,021 | $783 | $1,012 |
| GA | $923 | $1,054 | $849 | $1,057 | $756 | $1,079 | $976 | $938 | $841 | $935 | $756 | $1,079 |
| HI | $553 | $903 | $615 | $962 | $568 | $993 | -- | -- | $530 | $976 | $497 | $993 |
| IA | $827 | $1,093 | $778 | $1,019 | $739 | $938 | -- | -- | -- | -- | $739 | $938 |
| ID | $690 | $847 | $812 | $998 | $762 | $1,020 | $677 | $765 | $796 | $971 | $751 | $1,011 |
| IL | $797 | $1,019 | $804 | $1,050 | $923 | $1,062 | $749 | $992 | $829 | $1,022 | $719 | $1,052 |
| IN | $527 | $865 | $579 | $873 | $667 | $940 | -- | -- | -- | -- | $667 | $940 |
| KS | $749 | $995 | $704 | $1,032 | $749 | $1,023 | $844 | $1,021 | $710 | $1,062 | $749 | $1,023 |
| KY | $563 | $920 | $583 | $973 | $611 | $1,023 | -- | -- | -- | -- | $611 | $1,025 |
| LA | $619 | $1,025 | $767 | $1,016 | $835 | $1,101 | -- | -- | -- | $1,042 | $938 | $1,120 |
| MA | $589 | $947 | $632 | $883 | $632 | $913 | $583 | $909 | $632 | $883 | $627 | $913 |
| MD | $556 | $842 | $555 | $857 | $570 | $859 | $584 | $906 | $613 | $901 | $571 | $859 |
| ME | $907 | $1,036 | $641 | $1,034 | $932 | $1,052 | $907 | $1,036 | $878 | $1,033 | $932 | $1,052 |
| MI | $679 | $955 | $697 | $972 | $741 | $981 | $815 | $981 | $719 | $1,000 | $687 | $1,007 |
| MN | $753 | $913 | $618 | $854 | $618 | $870 | $882 | $956 | $759 | $896 | $553 | $875 |
| MO | $783 | $982 | $811 | $1,083 | $796 | $1,089 | $992 | $1,059 | $910 | $1,018 | $832 | $1,096 |
| MS | $531 | $931 | $780 | $1,045 | $915 | $1,050 | $557 | $931 | $780 | $1,045 | $915 | $1,050 |
| MT | $759 | $977 | $768 | $1,036 | $706 | $992 | $564 | -- | -- | -- | $899 | $993 |
| NC | $892 | $1,146 | $954 | $1,170 | $925 | $1,126 | -- | $1,090 | $954 | $1,170 | $953 | $1,128 |
| ND | $566 | $840 | $597 | $905 | $507 | $874 | $569 | $870 | $664 | $880 | $507 | $874 |
| NE | $830 | $1,091 | $703 | $1,031 | $658 | $1,004 | -- | -- | -- | $1,037 | $658 | $1,004 |
| NH | $592 | $985 | $622 | $948 | $938 | $957 | $727 | $1,007 | $693 | $958 | $938 | $957 |
| NJ | $866 | $995 | $791 | $929 | $929 | $998 | $893 | $988 | $908 | $934 | $929 | $998 |
| NM | $668 | $988 | $577 | $1,017 | $654 | $981 | $859 | $1,009 | $730 | $1,074 | $518 | $1,011 |
| NV | $563 | -- | $624 | $1,039 | $666 | $1,043 | $600 | $858 | $879 | $1,039 | $779 | $1,016 |
| NY | $857 | $1,117 | $950 | $1,125 | $941 | $1,167 | $850 | $1,025 | $915 | $1,041 | $919 | $1,167 |
| OH | $638 | $917 | $697 | $978 | $772 | $1,015 | $687 | $895 | $679 | $963 | $688 | $1,018 |
| OK | $735 | $1,017 | $793 | $1,039 | $743 | $1,064 | $557 | -- | $736 | $1,029 | $743 | $1,067 |
| OR | $569 | $904 | $630 | $964 | $681 | $990 | $584 | $929 | $637 | $982 | $687 | $989 |
| PA | $732 | $898 | $703 | $953 | $724 | $992 | $688 | $872 | $719 | $940 | $725 | $992 |
| RI | $586 | -- | $609 | -- | $624 | -- | $580 | -- | $585 | -- | $624 | -- |
| SC | $521 | $879 | $642 | $969 | $603 | $971 | $521 | $879 | $642 | $969 | $603 | $971 |
| SD | $698 | $966 | $724 | $996 | $729 | $1,036 | $644 | -- | -- | -- | $729 | $1,036 |
| TN | $748 | $1,031 | $787 | $1,024 | $772 | $1,018 | -- | $927 | $873 | $1,058 | $817 | $1,024 |
| TX | $678 | $993 | $733 | $983 | $870 | $998 | $712 | $1,007 | $707 | $938 | $798 | $987 |
| UT | $1,042 | $1,061 | $904 | $1,117 | $933 | $1,120 | $1,166 | -- | -- | $1,099 | $707 | $1,109 |
| VA | $613 | $1,066 | $738 | $1,075 | $729 | $1,040 | $575 | $1,013 | $745 | $1,113 | $745 | $1,042 |
| VT | $1,014 | $1,037 | $1,036 | $1,120 | $1,173 | $1,143 | $1,083 | -- | $1,121 | -- | $1,082 | -- |
| WA | $634 | $890 | $697 | $956 | $642 | $946 | $610 | $867 | $660 | $941 | $672 | $946 |
| WI | $749 | $974 | $792 | $1,014 | $787 | $1,016 | $896 | $1,020 | $747 | $1,046 | $747 | $1,038 |
| WV | $587 | $1,034 | $686 | $1,145 | $772 | $1,130 | -- | -- | $1,065 | $1,185 | $772 | $1,130 |
| WY | $853 | $1,065 | $713 | $1,088 | $725 | $1,099 | -- | -- | -- | -- | $725 | $1,099 |

*Abbreviations:* EHE, “Ending the HIV Epidemic”; QHP, Qualified Health Plan; DTG/ABC/3TC, dolutegravir/abacavir/lamivudine; BIC/FTC/TAF, bictegravir/emtricitabine/tenofovir alafenamide fumarate *Footnote:* All QHPs providing coverage were included in cost calculations, with exclusion of QHPs not providing coverage.
